# Progress in Perovskite Solar Cells towards Commercialization—A Review

**DOI:** 10.3390/ma14216569

**Published:** 2021-11-01

**Authors:** Hongqiao Wang, Yunfan Wang, Zhipeng Xuan, Tingting Chen, Jingquan Zhang, Xia Hao, Lili Wu, Iordania Constantinou, Dewei Zhao

**Affiliations:** 1 College of Materials Science and Engineering & Institute of New Energy and Low-Carbon Technology, Sichuan University, Chengdu 610065, China; 2019223010007@stu.scu.edu.cn (H.W.); 2018226220003@stu.scu.edu.cn (Z.X.); chentingting1@stu.scu.edu.cn (T.C.); zhangjq@scu.edu.cn (J.Z.); wulili@scu.edu.cn (L.W.); dewei.zhao@scu.edu.cn (D.Z.); 2 Department of Materials Science and Engineering, City University of Hong Kong, Kowloon Tong 999077, Hong Kong, China; yunfawang2-c@my.cityu.edu.hk; 3 Engineering Research Center of Alternative Energy Materials & Devices, Ministry of Education, Chengdu 610065, China; 4 Institute of Microtechnology (IMT), Technische Universität Braunschweig, Alte Salzdahlumer Str. 203, 38124 Braunschweig, Germany; i.constantinou@tu-braunschweig.de; 5 Center of Pharmaceutical Engineering (PVZ), Technische Universität Braunschweig, Franz-Liszt-Str. 35a, 38106 Braunschweig, Germany

**Keywords:** perovskite solar cells (PSCs), power conversion efficiency (PCE), fabrication technique, commercial promotion

## Abstract

In recent years, perovskite solar cells (PSCs) have experienced rapid development and have presented an excellent commercial prospect as the PSCs are made from raw materials that are readily and cheaply available depending on simple manufacturing techniques. However, the commercial production and utilization of PSCs remain immature, leading to substantial efforts needed to boost the development of scalable fabrication of PSCs, pilot scale tests, and the establishment of industrial production lines. In this way, the PSCs are expected to be successfully popularized from the laboratory to the photovoltaic market. In this review, the history of power conversion efficiency (PCE) for laboratory-scale PSCs is firstly introduced, and then some methods for maintaining high PCE in the upscaling process is displayed. The achievements in the stability and environmental friendliness of PSCs are also summarized because they are also of significance for commercialization. Finally, this review evaluates the commercialization prospects of PSCs from the economic view and provides a short outlook.

## 1. Introduction

**Figure 1:**
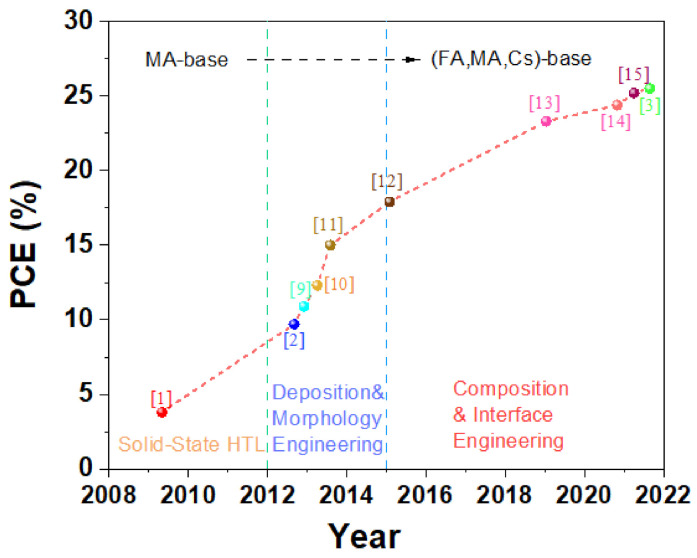
PCE development process of PSCs [1,2,3,9,10,11,12,13,14,15].

In recent years, organometallic halide perovskite solar cells (PSCs) have received significant attention from the photovoltaic community. In 2009, for the first time, CH_3_NH_3_PbI_3_ (MAPbI_3_) and CH_3_NH_3_PbBr_3_ (MAPbBr_3_) were introduced as photosensitizers in dye-sensitized solar cells and thus enabled efficiencies of 3.8% and 3.1%, respectively (Figure 1) [1]. In 2012, the efficiency was increased to 9.7% by replacing the liquid electrolyte with solid-state hole-transporting material (Spiro-OMeTAD) [2], with a profound influence on the development of high-efficiency PSCs. Based on this innovation, an impressive increase in efficiency was witnessed, and the highest efficiency of PSCs has even reached 25.5% so far (Figure 1) [3]. After increasingly intensive research was conducted, the merits of organometallic halide perovskite gradually appeared, including (1) an excellent light absorption coefficient (~10^5^ cm^−1^) and a tunable bandgap [4,5], (2) relatively low exciton activation energy (E_b_ ≈ 19 meV) [6], (3) long carrier diffusion length (>1 μm for MAPbI_x_Cl_3−x_) [7], and (4) excellent defect tolerance ability [8]. Accordingly, it is possible to obtain high-efficiency solar cells by combining the excellent light absorption capacity and effective transport of photo-generated carriers.

For popularizing the PSCs from the laboratory to the photovoltaic market, the rigid demand is to promote large-area PSC development. Large-area PSCs suffer an obvious decrease in the power conversion efficiency (PCE) due to the device nonuniformity, higher ohmic loss, and active area loss from interconnection. Common knowledge supports the optimization of the precursor solution composition, manufacturing method, and integration technology as the key to upscale photovoltaic devices. So far, the PCE of perovskite solar module (PSMs) is still inferior to that of laboratory-scale devices, resulting in the demand for further exploration and consideration of PSMs.

On the other hand, the poor long-term stability of PSCs remains the biggest obstacle to its industrialization process. Basically, the device stability tends to be affected by two aspects: (1) the irreversible degradation of the perovskite layer caused by an inappropriate environmental factor, the organic molecule volatilization [16], the O_2_/H_2_O permeation [17], high temperature [16], etc.; (2) the device instability that is related to the charge transfer materials [18], the absorption materials [19], and the electrode materials [20]. In order to overcome the PSC degradation and thus enable the PSCs to be industrialized, it is essential to concentrate on the material selection, device design, and interface modification. The use of water-soluble lead (Pb), toxic, capable of penetrating skin, and cancerogenic solutions also hinders the large-scale application of PSCs.

Given that challenges still exist in the PSC industrialization process, this review was performed and mainly contains (1) recent achievements in highly efficient PSCs with different areas, (2) the fabrication and optimization method in the upscaling process of PSCs/PSMs, (3) an overview of device stability, inevitable safety, and economic problems during the PSC commercialization process, and (4) the possible solutions to the Pb-leakage threat and the cost of device preparation as well as material recycling technology. Hopefully, this review is helpful in offering a comprehensive introduction to PSC devices and in deepening the knowledge on PSC utilization in a commercial manner.

## 2. Improved Performance of Large-Area PSMs

**Figure 2:**
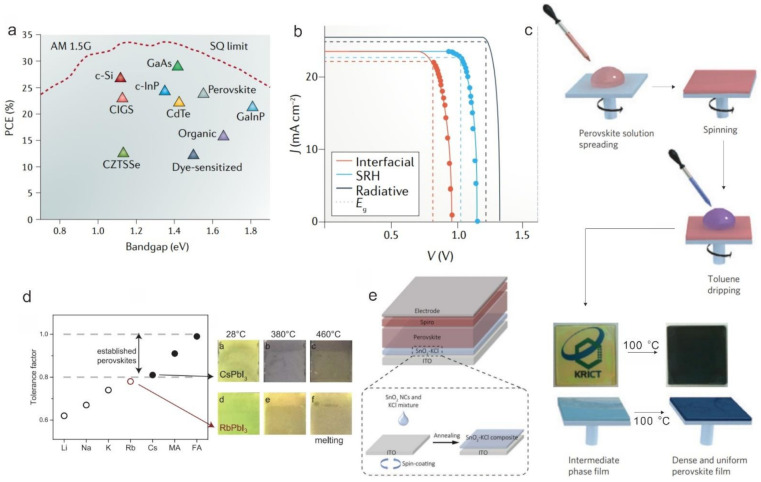
(**a**) PCE limit as a function of the bandgap for single junction solar cells calculated using the Shockley–Queisser (SQ) theory [25]. (not fully updated) (**b**) *J-V* curves for PSCs with different carrier recombination mechanisms [24]. (**c**) Solvent engineering procedure for preparing the uniform and dense perovskite film [26]. (**d**) Tolerance factor and perovskites at different temperatures with Rb doping [27]. (**e**) Device structure of planar PSCs and schematic fabrication process of SnO_2_-KCl composite ETL [31].

In only a decade, the PCE of PSCs has markedly increased to more than 25% from less than 4% [1,2,3,21,22,23], and thus PSCs have been emerging as a competitor to crystalline silicon solar cells and cadmium telluride solar cells [22]. As shown in Figure 2a, organometallic halide perovskites have a suitable bandgap, and the theoretical PCE of PSCs with a bandgap of 1.6 eV is 30.5%, according to the Shockley–Queisser limit [24]. However, this theoretical PCE only considers the radiative recombination without Shockley–Read–Hall (SRH) charge-carrier recombination and interfacial recombination, so the short circuit current density (*J*_SC_) and open circuit voltage (*V*_OC_) tend to further decline in practical devices (Figure 2a,b) [24,25]. Grain boundaries, ion, and interface defects are the main reasons for nonradiative recombination, which can be resolved by solvent (antisolvent) engineering, compositional engineering, or defect passivation, etc. In 2014, toluene drop-casting was innovatively introduced to prepare perovskite layers by Jeon et al.; the resulting perovskite films were extremely uniform and dense compared to other methods (Figure 2c). Due to the superior properties of the absorber, PSCs with a certified PCE of 16.2% without hysteresis were successfully fabricated [26]. Saliba et al. incorporated rubidium cations into perovskite to improve the perovskite material properties (Figure 2d) and reduced bulk recombination losses, where a high PCE of 21.6% with an excellent *V*_OC_ of 1.24 V was achieved [27]. In addition, Jiang et al. adopted phenethylammonium iodide (PEAI) to passivate perovskite film and aimed to reduce the surface defect, which enabled a high *V*_OC_ of 1.18 V that is close to the Shockley–Queisser limit *V*_OC_ (this composition is 1.25 eV) [13]. According to these three works mentioned above, the PCE of PSCs has been significantly improved relying on the fabrication method, compositional engineering, and interface engineering, providing new insights for other researchers in similar fields.

As for the PSCs, the performance improvement is also related to the optimization of the charge transport layers [18,28,29]. An ideal transport layer, usually containing a high carrier mobility, suitable band alignment, and high-quality film, has the ability to efficiently extract the carrier from the absorption layer and convey it to the front electrode. In view of this phenomenon, many efforts have also been made, such as optimization of the annealing process, doping engineering, and interface passivation. For example, Anaraki et al. investigated Nb doping of SnO_2_ deposited by a chemical bath deposition method, by which the roughness of the SnO_2_ surface was notably decreased, the FF was increased from 72% to 74%, and the hysteresis behavior in their corresponding devices was suppressed [30]. In addition, a SnO_2_-KCl composite electron transport layer (ETL) was employed in planer PSCs to simultaneously passivate the defects at the interface (ETL/Absorber layer), and this contact could be passivated by K and Cl ions (Figure 2e). A PCE increasing from 20.2% to 22.2% was obtained for the devices using such a composite ETL [31]. Although these mentioned highly-efficient PSCs are obtained based on a small scale (<1 cm^2^), they provide helpful insight that is the basis for scaling up technologies under the background that efficiency loss is a normal phenomenon when the devices are scaled up.

### 2.1. Scalable Coating Methods

**Figure 3:**
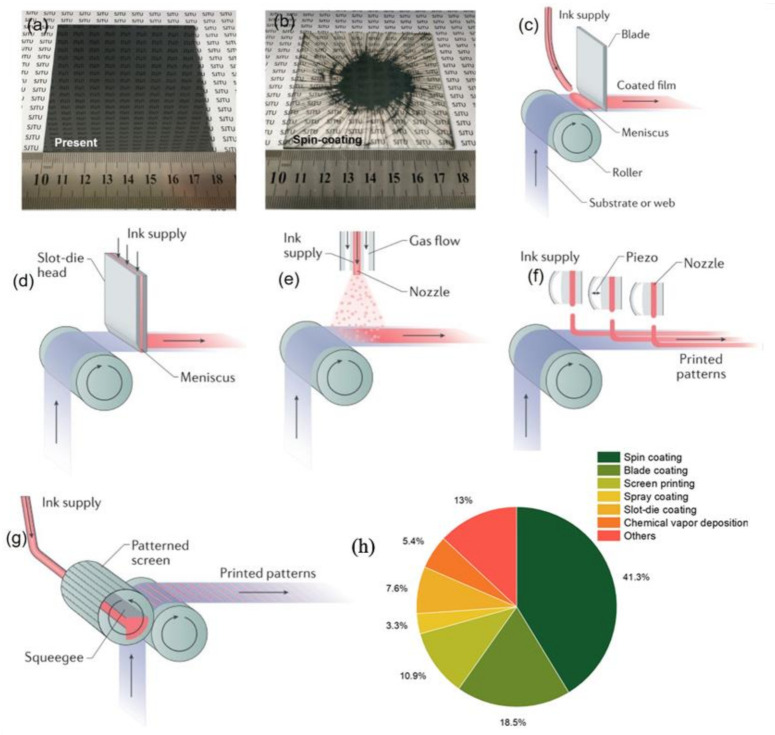
(**a**,**b**) Illustration of perovskite films deposited by the pressure-assisted processing method and by the spin-coating method [33]. (**c**) Blade-coating method [36]. (**d**) Slot-die coating method [36]. (**e**) Spray-coating method [36]. (**f**) Inkjet printing method [36]. (**g**) Screen printing method [36]. (**h**) Pie chart of the methods in the PSM fabrication (Data sources from Table S1) (not fully updated).

As aforementioned, the area of highly efficient PSCs is less than 1 cm^2^, far less than the required area for the commercialization of solar cells, while the state-of-the-art large-area PSM is 802 cm^2^, with a PCE of 11.6% [21,32]. Herein, the main reason of the efficiency gap between small area devices and large-area modules is the unsatisfactory large-area film of each layer after the scaling up of devices. Because the efficiency is of significance to push forward the commercialization of a solar cell, it is crucial to minimize the PCE loss while scaling up the PSCs. As for this issue, the fabrication of large-area functional layers is recommended, which has excellent uniformity and comparable optical–electrical properties to the small-area devices. The traditional fabrication methods such as spin-coating are generally limited to a square area (<10 × 10 cm^2^) in, which more than 90% of the precursor solution is wasted (Figure 3a,b) [33]. Moreover, with the increased area, the homogeneity of films fabricated by spin-coating decreases significantly, which results in a high process reliability and the acquisition of devices with good performance to be quite challenging [34,35,36]. Thus, further exploration of suitable coating methods is crucial for scaling up PSCs. In this work, the regular coating methods for large-area thin film deposition are summarized.

#### 2.1.1. Blade Coating

Blade coating, also named doctor blading, is one of the extensively used methods to fabricate scalable perovskite thin film. The precursor ink is flatted into a thin film by a blade on a smooth substrate, and then the wet thin film is dried to form a solid thin film (Figure 3c). The film thickness is generally controlled by several factors, such as the concentration and dispersion of the precursor ink, the working speed of the blade, the distance between the blade and substrate, and the temperature of the substrate [36]. By adjusting the initial ink thickness and the solvent evaporation rate, films of different thicknesses can be prepared, and the ink waste is substantially reduced compared with the traditional spin coating method [37,38,39,40,41,42,43,44]. Recently, Zhang et al. adopted this method to achieve a high PCE of 16.54% for 5 × 5 cm^2^ PSMs [45].

#### 2.1.2. Slot-Die Coating

The working unit of slot-die coating is a mechanically made fluid-die, where one side is connected to the pump to extract the precursor ink, and the other side with microfluidic metal die is the outlet of the precursor ink to form uniform wet films [36,46,47,48,49,50,51,52,53] (Figure 3d). Compared with blade-coating, this method has higher control accuracy and better repeatability, but it also requires higher quality and larger quantities of ink. As a result, slot-die coating has been less employed in solar module fields, and only limited reports are related to the film fabricated by slot-die coating. Recently, depending on the slot-die coating method, PSCs of 12 cm × 12 cm with a PCE of 14.3% have been fabricated, and uniform perovskite thin films of 80 cm × 80 cm have been prepared by the same process [54].

#### 2.1.3. Spray Coating

This method uses nozzles to spray highly dispersed droplets on the substrate in, which both the solvent evaporation and the crystallization rate are characterized as high on the high-temperature substrate [19,36,55,56,57,58,59,60,61,62,63,64] (Figure 3e). According to the generation modes of the droplets, the spraying method contains flow air-assisted spraying (through fast flow air), ultrasonic-assisted spraying (through ultrasonic dispersion), and electro-spraying (through electrical repulsion) [36].

However, as for this method, the balance between solvent evaporation and material redissolution (deposited materials can be dissolved again by new droplets) needs to be controlled carefully. Accordingly, this spray-coating method involves a more complicated fabrication process compared with blade coating. Recently, Taheri et al. employed sprayed SnO_2_ as the ETL and achieved a maximum PCE of 8.15% on an aperture area of 17.24 cm^2^ [65]. However, the coverage of droplets is random, so that several spray cycles are set to obtain a compact film.

#### 2.1.4. Inkjet Printing

Inkjet printing is a technique with multiple nozzles working simultaneously [17,20,36,66,67,68,69,70], as shown in Figure 3f. Each nozzle has its own ink supply channel and pressure control unit on the side to control the injection speed and volume of the “jet”. There are few reports about fabricating PSCs in this way, and whether it is suitable for the commercial process needs further investigation [36].

#### 2.1.5. Screen Printing

In screen printing, the printing ink is fixed with a reticulated net and is transferred to the substrate [71,72,73,74]. Excess areas of the screen are blocked by exposed photosensitive polymer emulsions, and as the rubber spatula spreads ink across the screen, the holes in the screen hold the viscous ink in place (Figure 3g) [36]. The thickness of the film depends on the mesh size and the thickness of the emulsion layer. Screen printing is usually used to fabricate thicker films (>1 μm) in PSCs [75]. This method is mainly used in devices with ZrO_2_ and carbon electrodes, by which Bashir et al. obtained a high PCE of 8.47% on a large aperture area of 70 cm^2^ [76].

In addition to these technologies described above, other deposition methods, such as physical vapor deposition (PVD) [77], chemical vapor deposition (CVD) [78], chemical bath deposition (CBD) [79], and co-sputtering etc. [80], are also used to fabricate large-area thin films.

In 2013, the deposition of perovskite thin films was first achieved using the PVD method [77]. Therein, the co-evaporation of PbCl_2_ and methylammonium iodide (MAI) generated uniform and compact MAPbI_3−x_Cl_x_ thin films, accordingly constructing efficient planar PSCs. However, the ultrathin (50 nm) and large-area (5 cm × 5 cm) perovskite films, usually difficult to be made using a solution method, were realized depending on the thermal evaporation [81].

The reaction of PbI_2_ thin films with MAI vapor was involved in the CVD, namely, the first method for the perovskite material deposition [82]. Afterwards, more CVD-based systems have been conducted under different conditions of chamber configurations and designs, reaction temperatures, and pressures, as well as sources of organic halide [83,84,85].

Basically, vapor-phase deposition is treated as a potential method to obtain perovskite thin films with large areas and thus is helpful in boosting the technology maturity. The vapor-phase deposition is solvent-free and accordingly is almost independent of the substrate, resulting in increasing attention paid to the continuous processing of tandem solar cells that are based on perovskite and are integrated with either low bandgap perovskites or available CIGS, Si, CdTe, etc. in commercial production. Nevertheless, these methods run with a requirement of complex apparatus and thus a high cost of device fabrication. Besides, a fabrication procedure meeting the ultra-high manufacturing capability (e.g., Si solar modules) remains challenging, which is the unique passage that is able to enable the Perovskite/Si tandem solar module to be cost competitive compared to the mature Si PV technology.

The common characteristic of these methods is the high cost and the requirement of complex apparatus, which increases the cost of device fabrication. From the data in the Table S1, Supplementary Materials (Reports on PSMs from 2014 to July 2021), we can obtain a pie chart (Figure 3h) of the methods used in PSM fabrication. From this chart, we can see that the spin-coating method is still the most common approach (accounts for around 40%), due to the simple and mature technology process of spin-coating. In addition, other scalable fabrication methods need further development.

### 2.2. Upscaling of the Absorber Layer

The fabrication of smooth and uniform large-area thin film is a huge challenge for scaling up the absorber layer. Perovskite crystals tend to have preferential growth and form dendritic structures in the natural drying process of the perovskite precursor solution, leading to numerous recombination centers and shunted paths to exist in the PSCs (cluster and pinhole) [86]. Thus, applying appropriate strategies to control crystal growth and to improve the large-area film quality is extremely urgent, which are summarized as follows.

#### 2.2.1. Compositional Engineering

**Figure 4:**
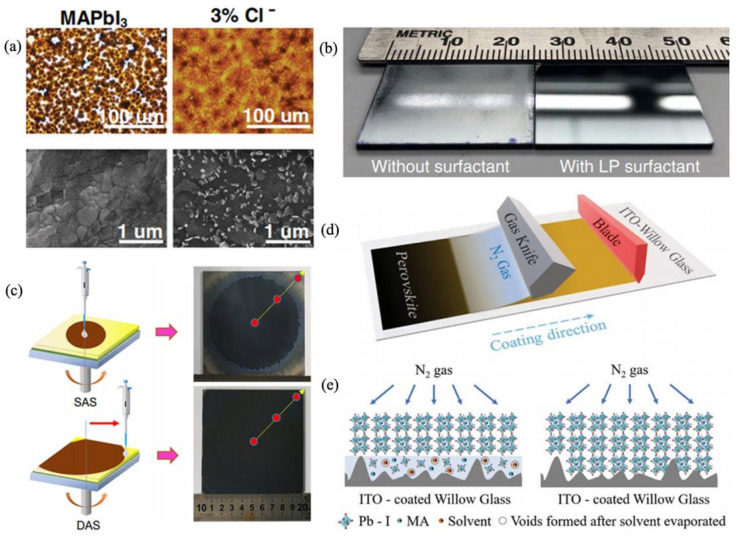
(**a**) Morphological characterization of perovskite MAPbI_3_ films without and with 3% Cl^−^ incorporation by optical microscopy and scanning electron microscopy [87]. (**b**) A photographic image of blade-coated perovskite films without and with LP surfactant [37]. (**c**) The schematic diagram of the static antisolvent process and dynamic antisolvent quenching process [100]. (**d**,**e**) Blade-coated perovskite films on indium tin oxide (ITO) coated with Willow Glass with N_2_ gas to improve film morphology [105].

The general perovskite formula is ABX_3_ in, which A is a monovalent cation (MA^+^, FA^+^ or Cs^+^), B is divalent metallic cation (Pb^2+^ or Sn^2+^), and X is a halide (Cl^−^, Br^−^ or I^−^). The properties of perovskite are tuned with different A, B, and X ions. Jeon et al. combined formamidinium Pb iodide (FAPbI_3_) with MAPbBr_3_ as the absorber materials and found that the film became more uniform and smoother with increasing Br concentration [12]. In addition, perovskite films obtained from a chloride-containing precursor with 3% Cl^−^ presented a better coverage (Figure 4a) [87]. Similarly, Qiu et al. added 60% PbCl_2_ into the mixed Pb source and fabricated a pinhole-free module with a PCE of 13.6% based on the above precursor [88]. Ren et al. used an LBIC image to confirm that PbI_2_ can improve the homogeneity of the perovskite film [23]. Generally, using Cl^−^ to replace I^−^ can suppress the formation of Pb-I-Pb plumbates and thus improve the morphology of thin films [89]. This compositional engineering provides some feasible ideas for scaling up the absorber layer.

As aforementioned, the extra Cl^-^ in composition is beneficial to film quality; likewise, its corresponding additives should be able to improve the film morphology. Jin et al. explored the partial substitution of PbI_2_ by ZnCl_2_ and its effect on perovskite morphology [90]. The introduction of ZnCl_2_ significantly increased the grain size and reduced the pinhole of perovskite film. This phenomenon is attributed to the formation of a chloride-containing intermediate phase, which postpones the nucleation and crystal-growth processes and thus enhances the coverage fraction of film. Ethlammonium chloride also was employed in combination with a facile solvent bathing approach to achieve high-quality MAPbI_3_ films, resulting in well-oriented and micron-sized grains and corresponding PSMs with a PCE of 7.36% (aperture area 25 cm^2^) [91]. Deng et al. found a slight excess of AX, where A is FA or the Cs cation, can improve the photostability by compensating iodide vacancies and suppressing ion migration and defect generation [16]. In addition, Lewis base, similar to dimethyl sulfoxide (DMSO) and N-methylpyrrolidone (NMP), is also a satisfactory additive to optimize the crystallization process of perovskite film. DMSO can be combined with strong Lewis acid (PbI_2_) according to Lewis acid–base theory. In this process, the nucleation and crystal growth rates can be affected by basicity; thus, whether in the field of organic or inorganic perovskite, DMSO is commonly used to improve the morphology of thin films. Via DMSO conduction, FAPbI_3_ perovskite showed better film quality, a larger grain size, and long-lived carrier lifetime, through, which a PCE of 19.7% with an aperture mask area of 0.125 cm^2^ was obtained [92]. In the scalable fabrication process, increasing the wettability of the perovskite solution on the substrate is essential for smooth film. Deng et al. showed that exceedingly small amounts of L-α-Phosphatidylcholine (LP) surfactant additives dramatically increased the adhesion of the perovskite ink to the underlying ETL, resulting in a PCE of 14.6% measured at an aperture area of 57.2 cm^2^. This surfactant could be a kind of general additive in perovskite ink to improve the perovskite film quality in various scalable fabrication methods (Figure 4b) [37].

Fabrication of 2D–3D hetero-structured perovskite is another way to control the perovskite film morphology. In 2018, Li et al. introduced a 2D C_6_H_18_N_2_O_2_PbI_4_ microcrystal into the precursor solution, the grain boundaries of the deposited 3D perovskite film were passivated, and less rough film was obtained by employing 2D perovskite. The grain size and surface coverage were increased with the enhancement of the 2D perovskite concentration, which was helpful to fabricate high-efficiency PSMs. Accordingly, PSCs with a PCE of 21.06% in small size and 11.59% in the solar module (active area 57 cm^2^) were obtained based on this 2D–3D phase-segregated vertical heterojunction [93].

#### 2.2.2. Solvent and Antisolvent Engineering

Although Cl^−^ is beneficial to improve the perovskite film morphology as mentioned above, directly obtaining a mixed halide perovskite thin film with a uniform composition is arduous due to the formation of unreacted MAI and MACl. Thus, Heo et al. adjusted the ratio of dimethylformamide (DMF) and γ-butyrolactone (GBL) to obtain the largest mixed halide perovskite crystal grains in the 8:2 ratio. In the fabrication process, underlying polycrystalline perovskite film with small crystal grains re-dissolved and merged into larger crystal grains by re-crystallization [59]. Similarly, Ozaki et al. introduced a high-purity MAPbI_3_ complex with intercalated DMF molecules as a precursor material for fabrication of dense perovskite film with the pure DMSO solvent, in addition, the low volatility of the pure DMSO solvent prolonged the time period for the antisolvent addition step. Then, the PSM with an optimized PCE of 11.5% was successfully achieved on the aperture area of 27.25 cm^2^ [94]. Li et al. added DMSO to 2-ME-based perovskite ink. By adjusting the amount of DMSO, they achieved 20.8% slot-die coated small area MAPbI_3_ PSCs [95]. Chiang introduced the H_2_O additive in the MAI/IPA solution, and high-quality film with a pure MAPbI_3_ phase, and larger grains were formed due to the higher penetration ability of MAI. Based on this investigation, controlling the humidity in the future fabrication process may also have the same effect [96].

Cooperation bonds between Pb ion and DMSO help to control the crystalline process of perovskite films. Inspired by this, Chen et al. employed crown ether as a Lewis base to further slow crystal growth, resulting in 4 cm × 4 cm PSMs with a PCE of 16.69% and excellent stability greater than 1000 h [97]. Novel forces such as hydrogen-bonding have attract researchers’ attention. Huang et al. introduced sulfolane to interact with organic cations by hydrogen-bonding forces, achieving a device efficiency of 16.06% with an active area of 36.6 cm^2^ [98]. Bai et al. introduced an ionic liquid, 1-butyl-3-methylimidazolium tetrafluoroborate (BMIMBF_4_), aiming to form halide complexes and to optimize the energy-level alignment, and accordingly observed PSCs with excellent stability greater than 1800 h under illumination at 70 to 75 °C [99].

The natural drying process is the main cause of the heterogeneous nucleation and crystallization of thin films. Antisolvent engineering has been applied to control halide perovskite material crystal growth, and it rapidly removes the solvent of thin films by coating, which could reduce the migration of the solute in the solution, thus generating a smoother and high-quality film. Toluene, chlorobenzene, and diethyl ether, etc., are common antisolvents with a strong solubility in solvents (DMF and DMSO). When the antisolvent was dripped into film surface, the solvent was quickly extracted. Bu et al. employed simple dynamic antisolvent quenching (DAS) to replace the traditional static antisolvent (SAS) process. This method provides a facile and universal approach to fabricate crack-free and uniform large-area perovskite films (Figure 4c) [100]. However, the present antisolvents used in the one-step spin-coating method always encounter problems with the narrow process window, which would limit the application of antisolvent in large-area film fabrication due to the delayed reaction. Zhao et al. introduced anisole into the one-step-coating method, and the dripping time ranged from 5 to 25 s. Finally, they achieved a high PCE of 17.39% for large-area (1.08 cm^2^) PSCs. These results provide a deeper understanding of antisolvent application in large-area PSMs [101].

#### 2.2.3. Physical Methods

Analogously, some physical approaches can also remove solvent rapidly and obtain high-quality films. For example, Chiang et al. adopted a hot solution and solvent annealing to fabricate perovskite film, where the film with casting engineering showed a higher grain size and less grain boundary compared with the control group, and a PCE of 14.3% for PSMs (active area of 25.2 cm^2^) was achieved [102]. Huang et al. adopted perovskite nanocapsules to promote homogeneous nucleation and achieved PCEs of 22.10% and 16.12% for PSCs and modules, respectively [103]. In addition, gas blowing was introduced by Gotanda et al., in, which N_2_ is blown onto the precursor solution after spin-coating and the substrate is then dipped in an antisolvent bath. Therein, the surface of perovskite fabricated with gas blowing showed a common dark-brown and uniform morphology, and a PCE of 14% for PSMs (active area of 25 cm^2^) was obtained [104]. Similarly, Dai et al. used N_2_ gas as the air knife. Moving the blade spreads the film across the substrate; an air knife moving with a blade simultaneously blows N_2_ gas on the as-coated wet film to remove the solvent, inducing crystallization (Figure 4d,e) [105].

### 2.3. Upscaling of the Transport Layer (ETL & HTL) and Back Electrode

Compared with charge transport materials in small-area PSCs, transport layers with a lower cost and more uniform film deserve more attention in large-area PSCs. As the traditional ETL, TiO_2_ is not suitable for widespread use in scalable PSCs due to its high temperature (>450 °C) fabricated process, which is also undesirable for wearable devices (large-area flexible device) [106]. Low-temperature solution fabrication with inexpensive materials can be widely used in future scalable produce. In comparison, SnO_2_ has gradually exhibited superiorities, which can be fabricated at a much lower temperature (<180 °C) [107]. Nevertheless, the SnO_2_-based ETL with spontaneous aggregation will form island morphology and local shunt pathways, which result in drastic nonradiative recombination [108]. Traditional HTL, such as Spiro-OMeTAD, has the same problem; thus, Qin et al. applied Bifluo-OMeTAD into slot-die coating to replace Spiro-OMeTAD, which can effectively suppress crystallization and improve film morphology [51]. Moreover, interfacial engineering is commonly used to solve interface defects and shunt pathways. A C_60_-self-assembled monolayer was introduced to passivate the surface of SnO_2_ [109]. With passivation, PSCs showed a significantly higher fill factor (FF), which demonstrate the shunt pathway was covered adequately and it led to decreased shunt resistance (*R*_SH_). Similarly, graphene could be used to “on-demand” tune the interface properties of PSCs. Agresti et al. on-demand modulated the photoelectrode charge dynamic by doping the mesoporous TiO_2_ layer with graphene flakes to optimize charge extraction, and they achieved a PCE of 9.2% based on a PSM with an aperture area of 69.52 cm^2^ [110].

In small-area PSCs, Au and Ag are common back electrodes adopted to form befitting energy band matches and to reduce contact resistance. However, due to the expensive price of Au and Ag, they must be substituted by other cheaper materials for reducing fabrication cost upon scaling up the size of devices, for example, the carbon electrode. Hu et al. employed a triple layer of mesoporous TiO_2_/ZrO_2_/carbon as a scaffold without any hole conductor or Au reflector. By optimizing the thickness of the tripe scaffold, PSMs (active area 49 cm^2^) with a PCE of 10.4% were obtained [111]. This paves the way for the awareness of efficient large-area PSMs for industrialization.

### 2.4. Module Design

Although large-area film with outstanding morphology has been fabricated, solar cells still struggle to achieve high PCE due to the parasitic resistance loss in the transparent conducting electrode, such as fluorine-doped tin oxide (FTO), ITO, Al-doped zinc oxide (AZO), and hydrogenated indium oxide [112]. Dividing a large-area PSCs into smaller sub-cells with series interconnection to form a module is needed.

#### 2.4.1. Fabrication of Series Connected Modules

**Figure 5:**
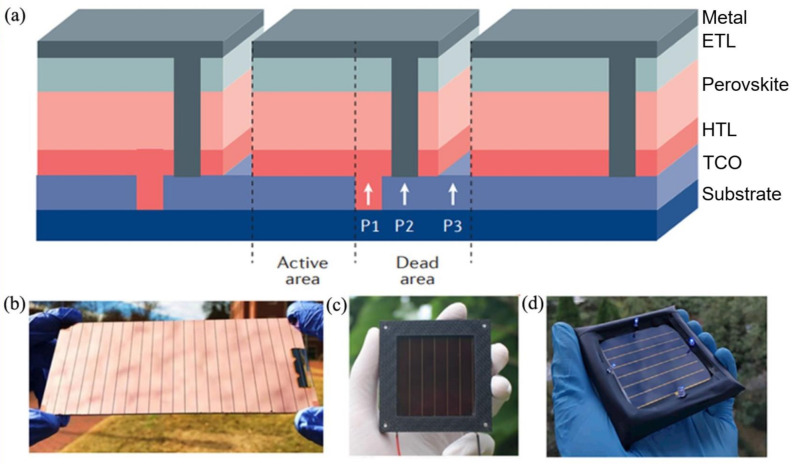
(**a**) Interconnection of a perovskite module fabricated by typical scribing processes for thin-film solar modules. [36] (**b**) In 2018, Deng et al. obtained PSMs with a PCE of 15.3% (aperture area 33 cm^2^) [37]. (**c**) In 2019, Qiu et al. obtained PSMs with a PCE of 12.3% (aperture area 22.8 cm^2^) [117] and (**d**) in 2020, Ren et al. obtained PSMs with a PCE of 17.88% (aperture area 25.49 cm^2^) [23].

Making a series-connected module is the most used interconnect approach, which is fabricated by a parallel scribe, and the laser or mechanical scribing can be used to finish this process. In general, the interconnection is composed of several parallel scribes referred to as P1, P2, and P3 (Figure 5a) [36]. In the first step, transparent conductive oxide on the glass substrate is divided into two parts by the P1 scribe to form stripe-shaped conducting electrodes. Secondly, after finishing the fabrication of the photovoltaic P1 layer (absorber and charge transport layer), the P2 scribe is added near the P1 scribe to connect the sub-cells in series. The P3 scribe, which is set to isolate the top electrode between neighboring cells, is parallel to the P1 and P2 and is added after deposition of the top contact [113]. The interconnection area is inactive to generate power and is a major source of power loss in PSMs, which is called the dead area. The ratio of the active area relative to the total area (sum of active area and dead area) of the module is named the geometric fill factor (GFF), which is a significant module parameter. A higher GFF of the module means less power loss for a given module size. To obtain such an excellent GFF, the scribes (P1, P2 and P3) should be narrow and placed as close together as possible. In the efficiency test process of solar cells, a shadow mask is commonly used to determine the illumination area, which is also defined as the aperture area. Applying the aperture area (including all interconnection areas) to calculate efficiency is well-accepted, on the contrary, applying the active area (without any interconnection area) could obtain a higher and incorrect efficiency due to the contribution of the dead area. In this case, it is necessary to present the GFF so that PCE of modules can be exactly compared.

#### 2.4.2. Optimization of Series-Connected Modules

The essential point for optimization of series-connected modules is to enhance the GFF (increase utilization ratio) and FF (decrease contact resistance) by minimizing the ratio of the dead area and optimizing interconnection technology (P2). Due to the advantages of a low cost, fast scribing rate, and high precision, laser scribing is commonly used in module fabrication, which can obtain a smaller interconnection contact resistance and prevent more waste area compared with the mechanical scribing. In 2017, a high GFF of 94% was obtained by Rakocevic, and this demonstrated that much less waste of PSMs can be achieved by mature laser patterning technology, and researchers can pay more attention to the decrease in contact resistance [114]. Recently, Ren et al. adjusted the strength of the laser and tried to avoid extra residue, in this case, they achieved a 25.49 cm^2^ (aperture area) PSMs with a 17.88%-certified efficiency and the highest FF (over 78%) in PSMs, and it also was the highest certified minimodule PCE in a recent report [23]. Considering the effect of residue (during P2 scribing) on the PSMs, choosing a flexible or soft-charge transport material is beneficial to remove the residue. In this case, Bu et al. used slot-die printing to fabricate a large size (5 cm × 6 cm) flexible module to obtain an efficiency greater than 15%; this flexible PSM had no hysteresis [115]. Moreover, soft-charge transport materials such as poly (triaryl amine) (PTAA) were employed on the polymer substrate and accordingly a record aperture efficiency of 15.86% on a flexible module (42.9 cm^2^) was obtained [105]. It is worth mentioning that, during the increase in the PSM area, increasing the length of the sub-cell can obtain a higher PCE than increasing the width, which could offer another design consideration for researchers in PSM fields [116]. Optimizing the sub-cell departure and module interconnection for PSMs is essential for further improving the module performance. Although an extremely large-area (802 cm^2^) module with 11.6% efficiency has been reported, the current condition of modules is still far from the normal scaling dependence [21], and most research has concentrated on the mini-module (the data in the figure were obtained from Table S1); some high-efficiency modules in previous work are displayed in Figure 5b,d.

In conclusion, various coating methods have been applied to the scalable process. In spite of various emerging fabrication techniques (PVD: 64 cm^2^, 15.8% [118]; CVD: 22.4 cm^2^, 12.3% [119]; blade coating: 57.8 cm^2^, 14.6% [37]; Slot-die coating: 149.5 cm^2^, 11.8% [47]; Inkjet printing: 198 cm^2^, 6.6% [120]), the most efficient PSMs with area >200 cm^2^ were successfully fabricated by using spin coating (12.6% with an aperture area of 354 cm^2^ on a 203 × 203 mm^2^ glass substrate) [121] and meniscus printing (11.6% with 802 cm^2^) [32]. Therefore, the printing-based methods seem to have potential in preparing scalable, low-cost PSMs. Methods such as blade-coating, spray-coating as well as screen printing will be further developed due to their low cost. Hence, it is essential for the commercialization of PSMs to pay more attention to these coating methods. On the other hand, high-efficiency PSMs must have a high GFF, which is closely related to the module design such as reducing the width of P1–P3.

## 3. Stability of PSCs

Recently, the PCE has reached >25% for single-junction devices [15]. Nevertheless, the poor long-term stability is still the main limitation for operational application, which has been one of the most critical development challenges of PSCs [122,123]. Apart from the instability caused by environmental issues such as high temperature, humidity, or light illumination, the inappropriate charge transport layers will not only impede the charge separation and transport but will also induce the degradation of the absorption layer [124]. Hence, many studies have enhanced the stability of the cell for industrial applications. In this part, we mainly summarize recent investigations on materials used in each functional layer for enhancing the stability of PSCs.

### 3.1. The Stability of the Hole Transport Layer

Organic Hole Transport Materials (HTMs) such as Spiro-OMeTAD and PTAA are commonly used in n-i-p structures. PEDOT:PSS can only be used in p-i-n structures. Indeed, Spiro-OMeTAD and PTAA with poor charge conductivity are not the most suitable HTMs. It is necessary to add tert-butylpyridine (t-BP) and lithium bis (trifluoromethanesulfonyl)imide (Li-TFSI) to enhance the performance of Spiro-OMeTAD and achieve higher PCE. Li-TFSI functions as the p-dopant to increase the hole conductivity and facilitate the oxidative reaction between Spiro-OMeTAD and O_2._ t-BP can enhance the hole extraction on the interface between the perovskite layer and HTL. However, Li-TFSI is hygroscopic and easy to liquefy, which will accelerate the degradation of the perovskite layer and reduce the stability of devices. Another additive (t-BP) can gradually evaporate at room temperature, leading to the formation of pinholes in the HTL, which makes the perovskite contact the mental electrode directly and induce a decrease in the *V*_OC_ of PSCs [125,126]. In addition, the acidic nature (pH < 1) and hygroscopicity of PEDOT:PSS could corrode electrode and induce the degradation of the perovskite layer when PEDOT:PSS contacts perovskite directly. The solvent of PEDOT:PSS is water, which has negative effect on the perovskite layer [127].

It is urgent to deal with these disadvantages of the frequently used organic HTLs. Several studies have been done to achieve superior stability of PSCs, such as to apply hydrophobic dopants, to introduce interlayers, and to adopt chemically stable HTMs.

#### 3.1.1. Organic HTMs

PEDOT:PSS with high transparency, high thermal stability, good mechanical flexibility, and a suitable band level is a commonly used HTM. Optimizations have been made to overcome the instability problem of PEDOT:PSS to further boost the long-term performance of PSCs.

**Figure 6:**
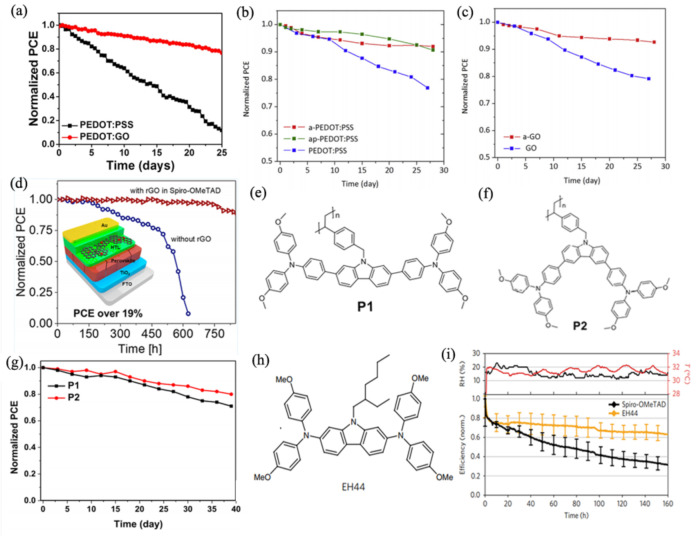
(**a**) Stability characteristics of the PSCs with PEDOT:PSS and PEDOT:GO composite films under ambient air conditions [42]; long-term stability result of devices with (**b**) PEDOT:PSS, a-PEDOT:PSS, ap-PEDOT:PSS [124], and (**c**) a-GO, GO [124], stored in N_2_ atmosphere; (**d**) the scheme of the structure of the device fabricated with rGO-added HTL and the stability test results [133]; the molecular structure of (**e**) P1 [134], (**f**) P2 [134]; (**g**) the stability test of devices with P1 or P2 [134]; (**h**) the molecular structure of EH44 [135]; (**i**) the operational stability test of TiO_2_/FAMACs/Spiro-OMeTAD or EH44/Au devices under ambient conditions [135].

Doping has proved to be an efficient method to regulate the pH value of PEDOT:PSS and enhance the stability of PSCs. Graphene oxide (GO) and its derivates with low cost and mild acidity are good alternatives to PEDOT:PSS. Yu et al. employed PEDOT:GO composite film as the HTM, and the devices exhibited a PCE of 18.09% with better environmental stability than devices based on PEDOT:PSS (Figure 6a). According to the report, this was due to the low acidity of the GO solution (pH 9), which suppressed the degradation of ITO and the perovskite layer [42]. The stability of devices was further improved by Wang’s group after treating GO and PEDOT:PSS with ammonia or ammonium to reduce the natural acidity of PEDOT:PSS. They presumed that the ammonium moieties of a-GO captured the I ions and restrained the layer-to-layer diffusion of I- ions. The long-term stability result shown in Figure 6b,c exhibited that non-corrosive HTMs had improved stability compared with pristine GO and PEDOT:PSS [124]. Other dopants, such as CuSCN [128,129] and Zn (TFSI)_2_ [130], were used to improve the crystallinity of perovskite and decrease the trap density, which could further enhance the resistance of water or oxygen.

In addition, modification of hygroscopic PSS could also make notable progress toward more stable HTM. Hu proposed water-rinsed self-assembled PEDOT:PSS and confirmed the PSS attached strongly onto ITO via In-O-S bonds. It appeared that more hydrophobic PEDOT distributed at the surface and formed an oriented arrangement with a stronger hydrophobic surface and less hygroscopic PSS component, which were beneficial to improve the moisture stability [70]. Lin et al. replaced PSS with a new dispersant sulfonated acetone–formaldehyde condensate (SAF) to obtain lower acidity and more hydrophobic HTM with pH value of 6, and SAF exhibited stronger absorption in the UV-visible region than PSS, which would help to suppress the decomposition of the perovskite layer caused by UV light [131].

Spiro-OMeTAD with Li-TFSI and tBP is another commonly used HTM in the n-i-p structure for high-efficiency PSCs. Liu et al. introduced an easy fabrication method to overcome the aggregation of Li-TFSI by introducing a small amount of PbI_2_ into Spiro-OMeTAD to form a complex (PbI_2_·X tBP), which could hinder the evaporation of tBP effectively and suppress the aggregation of Li-TFSI. The excess PbI_2_ also worked as an interlayer passivator to reduce the defect density. The devices based on modified HTM exhibited superior long-term stability than the control device in air under relative humidity: 20–30% or at 60–65 °C in a N_2_ atmosphere [132]. Guo et al. employed reduced graphene oxide (rGO) to modify Spiro-OMeTAD and confirmed the rGO could provide adsorption sites for Li^+^ ions and limit Li^+^ ion migration. The rGO-based device exhibited a drop of less than 3% of its initial PCE when stored at 25 °C under humidity of 40% for 700 h, while the pristine devices dropped to 75% of the initial value after 450 h and dropped to 0% after 600 h (Figure 6d) [133].

The majority of PSCs with high PCE still use conventional organic HTLs; however, the instability of the devices resulting from additives cannot be ignored. Wu et al. designed and synthesized two new dopant-free polymers as HTMs by attaching a carbazole-based hole-transporting unit to a polystyrene chain (Figure 6e,f) [134]. The polymer P2 with the triphenylamine substituent at the 3,6-positions of the carbazole unit achieved a PCE of 18.45%. However, two novel side chain polymers both showed excellent long-term stability; the PSC devices without encapsulation retained more than 80% of the initial PCE after 30 days in an ambient environment with approximately 30% humidity (Figure 6g) [134]. The application of Li^+^-free hydrophobic EH44 has been used as a more reliable HTM. Dr. Jeffrey used AgTFSI to oxidize EH44 (Figure 6h) and formed EH44^+^TFSI^−^ (EH44-ox). By blending different ratios of neat EH44 and EH44-ox to optimize the device performance, the improved EH44-based devices showed obvious enhancement in moisture stability tests (Figure 6i) [135].

#### 3.1.2. Inorganic HTMs

Inorganic HTMs are superior in chemical stability and have hole mobility, easy synthesis, and lower cost than organic HTMs [104,136,137,138,139].

Stoichiometric NiO is insulating, but the undoped NiO_x_ is well-known as a p-type semiconductor due to the Ni vacancies. NiO_x_ with an appropriate work function and high hole mobility is one promising HTM, whereas several limitations hinder the application of NiO_x_. The major factors are the unmatched band alignment and the low conductivity, which will result in hole accumulation at the perovskite interface and increase the charge recombination rate. Doping and surface treatment (e.g., oxygen plasma treatment) are useful methods to enhance the conductivity and regulate the WF to mitigate these problems. Metal ions such as Ag^+^, Cu^2+^, Zn^2+^, Cs^+^, and Y^3+^ can be used as effective dopants to improve the conductivity of NiO_x_ and hole extraction [104,136].

Cu is considered to occupy Ni sites and increase both carrier concentration and carrier mobility. Chen and coworkers prepared Cu-doped NiO_x_ nanoparticle ink to form high-quality films, and the Cu:NiO_x_-based devices retained ≈95% of initial PCE (stored under ambient conditions at 50–65% humidity) after 1000 h [137]. Yao et al. applied a bilayer structure of mesoscopic Cu:NiO_x_ /blocking layer-NiO_x_ to achieve more stable solar cells with improvements in the photocurrent and fill factor. The mp-Cu:NiO_x_ nanoparticles formed a highly porous surface area and reduced the recombination loss; simultaneously, the Cu:NiO_x_ bilayer-based cells exhibited superior light long-term stability (retained >90% of the initial PCE after 1000 h under light illumination without encapsulation in a dry cabinet at 25 °C and with <30% relative humidity) [138].

**Figure 7:**
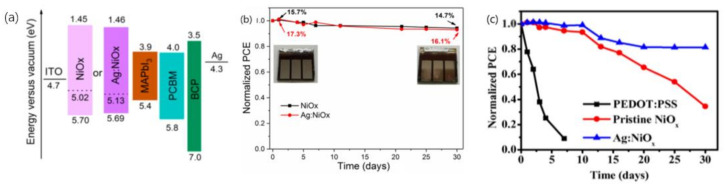
(**a**) Energy level diagram of PSCs with NiO_x_ and Ag:NiO_x_ HTLs [29]. (**b**) Stability test of the of PSCs based on NiO_x_ and Ag:NiO_x_ (5 mol %) as a function of storage (N_2_ box in the dark) time [29], (**c**) Stability test of PEDOT:PSS-based devices, NiO_x_-based devices and Ag:NiO_x_ (2 at.%)-based devices [139].

Silver ions are another effective p-type dopant for NiO_x_. Appropriate concentrations of Ag ions can effectively enhance the optical transparency, work function, electrical conductivity, and hole mobility of NiO_x_. Zheng’s group added 5 mol % Ag to NiO_x_ and confirmed that slightly incorporation of Ag^+^ doping would not degrade the performance of devices and could modify the energy alignment (Figure 7a). The control devices and Ag-based devices both retained 93% of the initial performance after 30 days of storage in a N_2_ box in the dark (Figure 7b) [29]. Wei et al. applied 2 at% Ag-doped NiO_x_ as HTL, and the Ag:NiO_x_-based devices exhibited higher environmental stability owing to the improved crystallinity and morphology of the perovskite layer formed on Ag:NiO_x_. The unencapsulated Ag:NiO_x_ cells maintained more than 80% of their initial PCE after one month of storage in the ambient environment (around 30% humidity, T = 25 °C) in a glass container (Figure 7c) [139].

Nevertheless, the doping of Cu ions will result in a decrease in transmittance and resistance under unsuitable Cu^2+^ concentrations. The incorporation of alkali ions, such as Li^+^, Na^+^, K^+^ or Cs^+^, in NiO_x_ can also achieve improvement in efficiency and stability of PSCs with negligible impact on the light absorption of NiO_x._ The suitable alkali ion dopants can optimize the carrier extraction ability and regulate the band level to reduce the band mismatch with perovskite layer so as to suppress the charge recombination. K^+^-doped NiO_x_ can not only improve the electrical properties but induce the formation of excess PbI_2_, which can further improve the surface or boundary passivation. Yin et al. proposed that the partial diffusion of K^+^ possibly replaced B sites and resulted in the increased amount of PbI_2_ [140]. Chen et al. demonstrated 19.35% using Cs-doped NiO_x_ as a hole extraction layer with inverted structure PSCs. The encapsulated Cs:NiO_x_ devices had excellent stability; the devices retained ≈90% of the initial efficiency after almost 80 days. The devices were tested in an ambient environment and stored in an argon glove box (H_2_O < 0.1ppm, O_2_ < 30 ppm) [141].

With the development of high-efficiency and stable PSCs, various materials have been studied as HTLs. Copper-based materials such as CuI, CuSCN, CuO_x_, and CuCaO_2_ and other inorganic materials are promising HTLs due to their photoelectrical characteristics.

**Figure 8:**
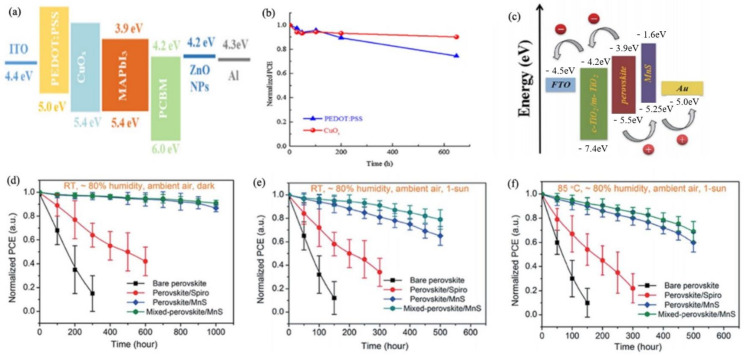
(**a**) The energy-level diagram of a typical device structure involving CuO_x_ [143]; (**b**) the stability test of PEDOT:PSS-based and CuO_x_-based devices, the normalized PCE decay of devices based on various HTLs [143]; (**c**)The energy-level diagram of a typical device structure involving MnS [144]; (**d**) under dark conditions [144]; (**e**) under continuous 1-sun illumination (100 mW cm^−2^) at room temperature in ambient air; [144] (**f**) thermal stability of PSCs at a maximum power point under continuous 1-sun illumination at 85 °C in ambient air [144].

CuI with a wide band gap of 3.1 eV were observed to improve the device stability due to its hydrophobic nature. Sun et al. achieved the highest PCE of 16.8% using room-temperature and solution-processed CuI as HTL, and this device maintained more than 93% of its original PCE after 288 h of storage under 25% humidity and room temperature [142]. CuO_x_ including Cu_2_O and CuO are typical p-type semiconductors and can work as attractive inorganic HTMs in PSCs due to their high conductivity, solution-processing, and suitable band levels (Figure 8a). [143] Yu et al. reported one inverted PSCs based on CuO_x_, which greatly improved the stability. The CuO_x_-based devices maintained 90% of the initial efficiency after 650 h, as shown in Figure 8b [143].

Novel, economical, and environmentally friendly MnS can be applied as an efficient HTL of PSCs. Li et al. prepared MnS by vacuum vapor deposition. MnS with a suitable band alignment (Figure 8c) and hydrophobic properties is beneficial to achieve high-efficiency and stable devices. The cells based on MnS achieved a champion PCE of 19.86% and had a superior stability over their counterparts adopting organic HTLs. MnS-based devices retained over 90% of their initial efficiency after exposure in air with a relative humidity of 80% for 1000 h without any encapsulation (Figure 8d). Remarkably, the devices based on MnS performed better with long-term stability under harsh conditions. Under 1-sun illumination, in the air with ~80% humidity the MnS-based devices could retain more than 80% of the initial PCE for 500 h at room temperature (Figure 8e) and retained 89% of the initial PCE after 400 h at 85 °C (Figure 8f) [144].

### 3.2. The Stability of the Electron Transport Layer

The stability of PSCs is strongly influenced by the properties of adjacent functional layers. Properly selected hole-transport and electron transport layers can provide an effective isolation for the perovskite layer and decelerate the degradation resulting from moisture and oxygen in the ambient environment. The most used ETL is TiO_2_ with n-i-p architecture and Phenyl-C_61_-butyric acid methyl ester (PCBM) with p-i-n architecture. TiO_2_ is known to be aggressive with respect to the perovskite layer, and the strong photocatalytic effect can reduce the stability of PSCs under illumination (including ultraviolet light). It is difficult for PCBM to form uniform and defect-free coatings on perovskite films. In addition, the aggregation behavior of PCBM and tendency to crystallize might form voids and pinholes in ETL, accelerating the degradation of the perovskite layer. The more severe problem is that PCBM facilitates photodecomposition of complex Pb halides by absorbing organic iodide (MAI) in the cavities of the crystal lattice. Therefore, several studies have investigated more suitable HTLs to enhance the PCE and stability of PSCs. Here, we outline the notable achievements of ETLs in recent years.

**Figure 9:**
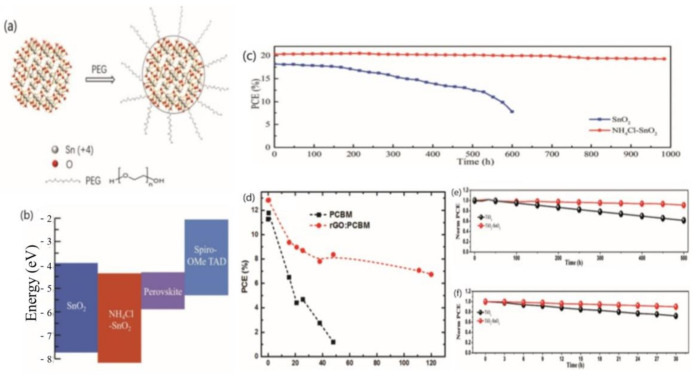
(**a**) Schematic diagram of the interaction between PEG and SnO_2_ [145], (**b**) the illustration of the energy band alignment of SnO_2_, NH_4_Cl-SnO_2_, and the perovskite layer [146], (**c**) the stability test of SnO_2_-based and NH_4_Cl-SnO_2_-based devices [146], (**d**) the stability test of PCBM-based devices and rGO:PCBM-based devices [147]; the stability test of TiO_2_-based devices and SnO_2_/TiO_2_-based devices (**e**) under continuous light illumination at room temperature [18], and (**f**) under continuous UV light illumination inside a dry air box [18].

To realize highly efficient and stable PSCs, a considerable number of studies have been carried out. SnO_2_ is one promising alternative due to its better photoelectric properties, excellent band alignment to the perovskite layer, and superior stability compared with conventional TiO_2_. Wei et al. incorporated polyethylene glycol (PEG) in the SnO_2_ ink (Figure 9a) to produce high-quality ETL; the device exhibited an obvious improvement in stability. In contrast to the fast PCE decay from 17.5% to 7% within 10 d of SnO_2_-based devices, PEG-incorporated SnO_2_-PSC maintained more than 97% of its initial PCE after a period of 90 d. The long-term stability tests of PSCs were examined under dark (30–80% RH) and under illumination without any encapsulation. The enhanced stability was mainly due to the improved bonding ability gained though the modification of PEG at the ETL/perovskite interface and the protection effect of PEG on perovskite at the interface [145]. In order to improve the electronic property of the SnO_2_ and passivate the interface between the perovskite and the SnO_2_, Liu et al. introduced ammonium chloride (NH_4_Cl) into commercial SnO_2_ aqueous colloidal dispersion. In this research, they observed an increase in electron mobility and a proper energy band alignment well-matched to perovskite (Figure 9b). Furthermore, NH_4_^+^and Cl^−^ effectively passivated the defects at the ETL/perovskite interface. Long-term stability was traced once a day with the PSCs stored in the glove box. The PSCs using the NH_4_Cl/SnO_2_ film as the ETL maintained more than 95% of their initial PCE after storage for 1000 h (Figure 9c) [146].

Fullerene and its derivatives are ideal alternative HTMs, but they suffer from chemical instability and low electron mobility [104]. George et al. introduced reduced graphene oxide (rGO) in PCBM to optimize the ELT/perovskite interface, passivate the top surface of the perovskite layer, and improve the grain size simultaneously. Compared with the control device, the rGO-based devices retained an almost fivefold higher PCE after ≈50 h of continuous solar illumination at high levels of relative humidity (RH) (>50%) (Figure 9d) [147]. Xu et al. found hydrophilic PCBB-OEG was an effective dopant for PCBM to boost the stability against oxygen. The PCBB-OEG doped devices retained 98.4% of the initial efficiency after 300 h storage in the ambient atmosphere without encapsulation [148].

Some research employed an interface layer between ETL and perovskite or HTL and the electrode in order to enhance the device stability under continuous light illumination. Mohammad provided an ETL, that is, an amorphous layer of SnO_2_ on top of the TiO_2_ compact layer fabricated by the solution process. The double layer of a-SnO_2_/c-TiO_2_ can improved the carrier transportation, band alignment, PCE, hysteresis, and stability of PSCs drastically. The stability results of devices after 500 h under continuous light illumination showed that a-SnO_2_/c-TiO_2_ device retained 91% of its initial PCE value, higher than that of the pure c-TiO_2_ device (67%) (Figure 9e,f) [18].

### 3.3. The Stability of the Perovskite Layer

Generally, the instability of PSCs originates from the interfaces and bulk of the perovskite absorber layer. Perovskites are solids with an ABX_3_ composition in which X is an anion and A and B are cations of different sizes (A is larger than B, e.g., A = CH_3_NH_3_^+^, CH(NH_2_)_2_^+^, Cs ^+^; B = Pb^2+^, Sn^2+^; X = Cl^−^, Br^−^, I^−^). The B and X ions are from BX_6_^4-^ octahedra; the A cation is located in the cavity between the four BX_6_^4−^ octahedra and forms a 3D periodic structure. The symmetry of the materials and the stability of the producing devices depend on the scale and shape of the lattice structure. A cation influences the crystal phase of perovskite directly, as the size of the A cations can causes shrinkage or expansion of the entire network. MAPbI_3_ was first used in photovoltaic devices, but it had poor stability under atmospheric conditions. Degradation induced by high temperature, oxygen, atmospheric water or light of MA-based perovskite has been widely studied. Partly or entirely substituting A cations is aimed to achieve a more stable perovskite crystal phase and promote the stability of perovskite films and devices. FA^+^ with a relatively larger radius is the most investigated organic cation alternate for MA. FAPbI_3_ has a higher tolerance factor (*t* = 0.88) than MAPbI_3_ and forms a cubic phase proved to be more stable than the tetragonal phase of MAPbI_3_. Numerous achievements indicate FA-based and MA-FA mixed perovskite display superior stability when exposed to moisture, elevated temperature, UV light, and so forth. Inorganic cations such as alkali metal ions are also suggested to replace organic A cations to improve the stability and performance of devices [149,150,151].

B cations are usually toxic and environmentally unfriendly Pb^2+^; other divalent cations such as Sn^2+^, [152] Ge^2+^, [153] Mn^2+^, [154] and Zn^2+^ [155] have been researched to replace Pb^2+^ to tune the bandgap and decrease the content of Pb^2+^. Perovskite with Sn^2+^ exhibits poor stability as Sn^2+^ is extremely easy to oxidize, which remains an intractable issue to be resolved.

Numerous studies on organic–inorganic hybrid materials, all-inorganic perovskite, have been performed to achieve higher thermal-, moisture-, and light-stability of PSCs. Here, we summarize the recent progress on the chemical composition, dopant engineering, surface or interface modification, encapsulation engineering, and other useful strategies for optimizing the stability of PSCs.

#### 3.3.1. Organic–Inorganic Hybrid Perovskites

Regulating the composition of perovskite and inserting a hydrophobic interlayer or other surface/interface engineering are commonly used to enhance the stability of PSCs. In the composition substitution field, A cations are commonly MA^+^, FA^+^, Cs^+^, Rb^+^, and K^+^, and B cations are divalent ions, e.g., Pb^2+^, Sn^2+^, Mn^2+^, Zn^2+^, Ge^2+^, etc.; the X anions tend to be I^−^, Cl^−^, Br^−^ or SCN^−^, SeCN^−^. By replacing or mixing the composition to achieve the promotion of stability mainly due to the improvement of perovskite film, one can suppress the ion migration or reduce the defect density.

**Figure 10:**
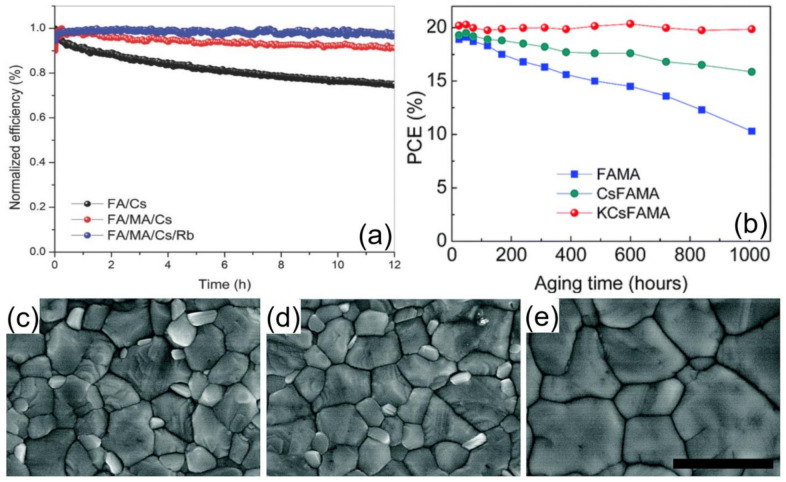
(**a**) Light stability of opaque perovskite cells on FTO substrates with different cation compositions upon 12 h under continuous 1-sun illumination [159]. (**b**) Long-term stabilities of the best perovskite devices stored under ambient air conditions without encapsulation for longer than 1000 h. [160] SEM images of (**c**) FAMA [160], (**d**) CsFAMA [160], and (**e**) KCsFAMA [160] perovskite films. The scale bar is 1 mm.

The small amount of excess PbI_2_ can work as a passivation agent, reducing charge recombination by formation of I-type band alignment, while a large amount of undesirable PbI_2_ is likely to be a recombination center and cause a decrease in the device stability [13]. Notably, high doping of Br^−^ and Cs^+^ will induce phase segregation, which relates to the halide migration through defects especially in the grain boundaries in a mixed-halide system. Duong et al. proved that appropriate doping of Rb^+^ could suppress the ion migration effectively and enhance the crystallinity with a larger grain size, finally improving the light stability performance of devices [156]. Matsui’s group demonstrated that the incorporation of Rubidium (Rb) cations into triple-cation perovskite (Cs_0.05_(MA_0.17_FA_0.83_)_0.95_Pb(I_0.83_Br_0.17_)_3_) caused the formation of RbPbI_3_, which can suppress the growth of PbI_2_. The devices were stored at 85 °C, under dry air (<5% humidity), and the devices with and without Rb retained 74% and 61%, respectively, of the initial PCE after 347 h. [157] Alkali metal ions such as K^+^, Rb^+^, and Cs^+^ have been fully researched to supersede the organic molecule to produce higher quality perovskite film with better crystallinity [157,158,159]. Considering of the tolerance factor, only Cs^+^ can be used to sustain the perovskite structure. Mixed cation PSCs showed better stability (Figure 10a). [159] Bu et al. compared the effect of Cs^+^−K^+^ cooperation and Cs^+^ alone on the device hysteresis and stability. Ultimately, KC_S_FAMA-cells exhibited the best stability (Figure 10b) and apparent increase in grain size (Figure 10c–e) compared with the CsFAMA and FAMA groups with a negligible decrease in PCE over 1000 h stored under ambient air (10 ± 5 RH%) without encapsulation [160]. Tong et al. introduced NH_4_Cl, which forms an intermediate phase with PbI_2_ to regulate the crystallization process, and fabricated 10 × 10 cm^2^ modules with 80% PCE after 1100 h under continuous light illumination [161].

Materials such as PEAI that contain hydrophobic long-chain organic cations as termination groups and can react with residual PbI_2_ in perovskite layers, forming wide-band gap 2D perovskites, which show better stability but poor PCEs than 3D perovskites, and can passivate the interface defects and block the water and oxygen into the perovskite layer to enhance the resistance to intrinsic and extrinsic degradation [162]. The reactions can be described as follows [163]:

$$2\mathrm{PEAI}+{\mathrm{PbI}}_{2}≜{\left(\mathrm{PEA}\right)}_{2}{\mathrm{PbI}}_{4}$$

$$2\mathrm{BAI}+{\mathrm{nPbI}}_{2}+\mathrm{nMAI}≜{\left(\mathrm{BA}\right)}_{2}{\mathrm{MA}}_{n-1}{\mathrm{Pb}}_{n}I_{3n+1}+\mathrm{MAI}$$

Li et al. introduced suit growth of gradient diffusion 2D pure PEA_2_PbI_4_ in 3D MAPbI_3_; both the 2D–3D mixed devices and 2D–3D gradient devices exhibited improved moisture stability after 15 days of storage in an air environment with 35~55% humidity, which could be due to hydrophobic benzene groups in PEA^+^, and the 2D–3D gradient devices showed excellent operation stability at maximum power point under AM 1.5G illumination with 100 mW/cm^2^ over 1000 h relative to the regular 3D PSCs [162]. You et al. reported another effective method in defect passivation, and they deposited organic halide salt PEAI, rather than 2D PEA_2_PbI_4_ perovskite, on the perovskite (FA_1−x_MA_x_PbI_3_) to suppress the surface defects and to decrease non-radiative recombination, by which great improvement of thermal stability was achieved. The PCE of the PEAI-treated device decreased at the start of several hours of annealing at 85 °C, and then the performance remained almost constant up to 500 h [12]. FAPbI_3_ is known to undergo phase conversion under ambient conditions, but the propriate 2D adducts can stabilize the cubic structure of FAPbI_3_. Lee et al. incorporated 1.67 mol % PEAI into the perovskite precursor solution to protect the FA-based perovskite from moisture and suppress ion migration; the prepared devices with efficiency above 20% maintained 98% of their initial PCE after 1392 h in dark [164].

**Figure 11:**
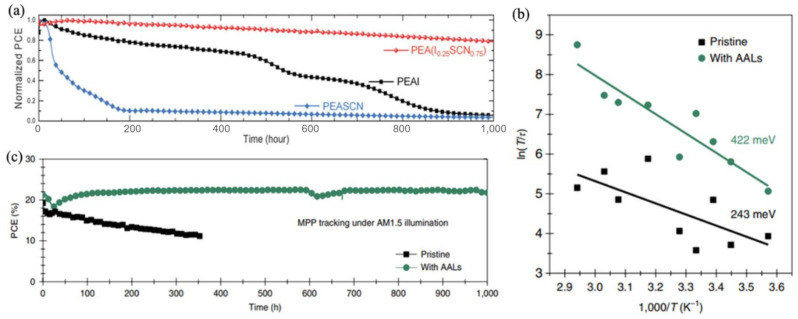
(**a**) Long-term stability of perovskite devices with different 2D additives under light illumination without encapsulation [165]. (**b**) Arrhenius plots (obtained by linear fitting of data points) of the temperature dependence of T/τ, showing an ion migration activation energy (E_a_) for pristine films and films with AALs, respectively [166]. (**c**) Long-term stability of the pristine CsFAMA device and the CsFAMA device with AALs under constant simulated solar illumination (100 mW cm^−2^) in a N_2_ atmosphere with a UV filter with a 420-nm cut-off [166].

Introducing both Pb(SCN)_2_ and PEAI to perovskite precursor to form 2D or quasi-2D materials is an efficient method to enhance the operational stability of PSCs. Kim et al. regulated the doping concentration of PEAI and Pb(SCN)_2_ and fabricated devices with outstanding stability that could maintain more than 80% of the initial PCE of 20.7% after 1000 h of continuous illumination [165], and other work based on the same dopants with different proportion showed <4% degradation under storage at room temperature in a N_2_ environment for longer than 4000 h (Figure 11a). [165] Zheng’s group studied the length of different surface-anchoring alkylamine ligands (AAL), and perovskite film with long-alkyl-chain AALs (OA or OAm) exhibited longer carrier lifetimes (Figure 11b) and larger water contact angles. The AAL-modified perovskite showed more n-types, and the ion migration was hindered, which were beneficial to the stability. The devices with AALs exhibited no PCE loss after continuous operation for 1000 h under AM1.5 illumination in a N_2_ atmosphere with a UV filter with a 420-nm cut-off, and lost only around 10% of their initial PCE after a thermal stability test of ~1020 h (T = 85 °C) (Figure 11c) [166].

Works on the substitution of Pb^2+^ to enhance stability have made some progress. Ion dopants (e.g., Zn^2+^, Mn^2+^) with a smaller ionic radius than Pb^2+^ can cause shrinkage of the crystal lattice, which may compensate the expansion result from the illumination or elevated temperature. The inclusion of appropriate concentration of Zn^2+^ or Mn^2+^ at the B site can reduce the void in the [BX_6_]^4−^ octahedral, enhance the interaction between the octahedral, and suppress the defect recombination, leading to a more stable crystal structure [102]. Liu et al. applied Mn-doped MAPbI_3_ to achieve high film quality, larger grain size, and reduced carrier recombination. The best devices with 1% MnI_2_ doping represented greater stability as the improved devices retained 95% of the original PCE while the control devices dropped to 87% (after storing under 30% humidity and 25 °C after 48 h) [167]. Li et al. fabricated Zn-doped MAPbBr_3_ single crystal (MPZB) with increased structure stability and lower density of defect states. It was confirmed that Zn^2+^ could be inserted into the perovskite lattice and reduce unwanted vacancy defects inside the octahedron. The Zn-doped devices showed a higher PCE of 19.09% with better thermal stability and excellent long-term photostability. Long-term photostability tests were operated by monitoring the PL intensity of perovskite films under illumination using a 325 nm continuous wave laser with power density around 70 mW cm^−2^, and the samples were exposed to laser light directly at room temperature for 4 h per day. The MPZB sample had negligible loss after 60 days, but the pristine samples decreased to ~40% under the same condition [168].

Other interesting works in boundary passivation through doping or molecular modification are also noteworthy. Qin’s group added core-shell Au@CdS nanoparticles into antisolvent in the perovskite-making process; the Au@CdS formed the intermediate Au@CdS–PbI_2_ adduct, which not only induced heterogeneous nucleation for high-quality perovskite film, but also adjusted the band alignment and decreased the barrier between HTL and the perovskite layer. The stability of Au@CdS-modified cells showed high stability due to the high quality of the perovskite film and the effect defensive function of H_2_O invasion [169]. Sha et al. sandwiched perovskite film and HTL with bridge-jointed graphene oxide nanosheets (BJ-GO) to immobilize iodide ions, passivate Pb defects, and tune surface energy [170]. Other dopants such as PBTI [171] and organic-conjugated molecules with rhodamine moieties (e.g., SA-1 and SA-2) [172] also work as effective passivators at the boundaries and boost the stability of devices. The stability of PSCs can be enhanced significantly by applying a customized thin-film encapsulation (TFE), which is composed of a multilayer stack of organic/inorganic layers that are deposited by chemical vapor deposition and atomic layer deposition (ALD). Lee et al. used low-temperature ALD to integrate TFE directly into functional layers to enhance the stability of PTAA-based PSCs. The multilayer structure showed excellent hydrophobic property and helped the devices maintain 97% of their initial efficiency after exposure to 50 °C and 50% RH for 300 h [173].

#### 3.3.2. All-Inorganic PSCs

The organic components are thermally unstable and easy to volatilize, resulting in the instability of the absorber layer. All-inorganic PSCs are developed due to the nonexistence of any volatile organic components and the better thermal stability.

CsPbI_3_, CsPbBr_3_, CsPbI_2_Br, and CsPbIBr_2_ are the most widely studied all-inorganic perovskites. CsPbI_3_ has a suitable band gap (1.73 eV), while CsPbIBr_2_ (≈2.05 eV), CsPbI_2_Br (≈1.91 eV) and CsPbBr_3_(2.3 eV) have large band gaps. High-quality CsPbX_3_ films are difficult to synthesize and undergo phase transition at room temperature and in a moist environment, that is, the desirable cubic perovskite phase spontaneously transforms to the orthorhombic, more thermodynamically stable σ phase. It is very desirable to design the component and ratio of all-inorganic halide perovskites to reach higher stability and efficiency [174].

Liang et al. designed Mn substitution in Cs-based Pb halide for high-stability all-inorganic PSCs. Mn doping modulated the electronic and optical properties of CsPbIBr_2_, and the Mn-doped perovskite film showed better crystallinity and morphology than the un-doped counterpart with an appropriate concentration of Mn dopants. The PCE of encapsulated PSCs based on non-doped CsPbIBr_2_ decreased by 20% of its initial value after about 144 h, while the PCE of encapsulated Mn-doped PSCs decreased by 8% after storage in the ambient environment for more than 300 h [175]. Tan et al. proposed Li-doped CsPbIBr_2_ with no-additive HTL-CuPc, which is highly hydrophobic, chemically stable, and beneficial to enhance the resistance to moisture of perovskite. The insertion of Li ions enhanced the interaction between Cs, Pb, and halides so as to boost highly stable chemical stability, and Li ions also helped to reduce the defect density and make smoother perovskite film [176]. Xiang et al. utilized Europium, which has been reported as an efficient sensitizer of photoluminescence in perovskites, to increase the tolerance factor of CsPbI_2_Br and result in an enhancement of the perovskite stability. The unencapsulated Eu-doped (CsPb_0.95_Eu_0.05_I_2_Br) device showed no signs of degradation during the first 300 h and retained 93% of its initial efficiency at the end of the test (370 h) under continuous white light LED illumination at 100 mW cm^−2^ with N_2_ gas flow [177]. Wang et al. introduced Zn(Ac)_2_ to CsPbI_2_Br to improve the thermal stability of CsPbIBr_2._ Partial replacement of Pb^2+^ with Zn^2+^ can increase the tolerance factor and stabilize the dark phase. The Ac^−^ group might passivate the defects at the boundaries. The devices based on Zn(AC)_2_ had better long-term stability than in dry air (15–20% humidity, 25 °C) due to the enhanced stable structure. The unencapsulated devices retained >90% of their initial PCE after 30 days [178].

### 3.4. The Stability of Electrodes

**Figure 12:**
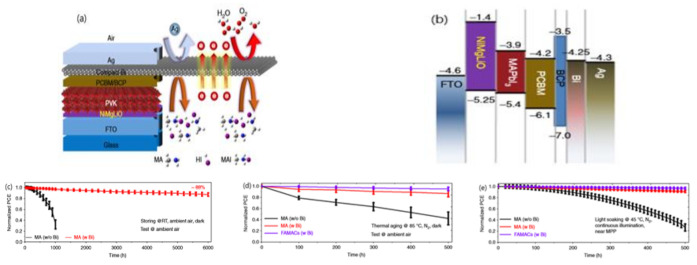
(**a**) Schematic diagram of the device structure; the Bi interlayer has a superior shielding capability, prohibiting both inward and outward permeation [179]. (**b**) Energy level diagram and diagram of the device [179], Stability test of unencapsulated devices (**c**) stored in the dark in ambient air at RT without humidity control, and J–V curves acquired periodically in ambient air [179]; (**d**) aged in the dark at 85 °C in a N_2_ atmosphere, and J–V curves acquired periodically in ambient air [179]; (**e**) aged under continuous illumination in a N_2_ atmosphere with electrical biases (0.641–0.885 V) near MPP at a cell temperature of 45 °C. The light intensity for aging was generated by a white light LED array and calibrated to achieve the same J_SC_ from the devices as for 1-sun AM1.5G solar irradiation [179].

Silver (Ag) and gold (Au) are widely used as electrodes in PSCs. Apart from the high material cost, using metal electrodes induces a metal ion-driven degradation in which metal ions on top migrate into the perovskite layer, lowering the PSC stability. Using interlayers to isolate the undesirable external moisture can protect the metal electrode from iodine corrosion. Wu et al. demonstrated a bismuth interlayer between the absorption layer and Ag electrode (Figure 12a,b) to enhance device stability. The Bismuth-interlayer-based devices exhibited greatly improved stability when subjected to humidity, thermal, and light stresses. The unencapsulated device retained 88% of its initial efficiency in ambient air in the dark for more than 6000 h; the devices maintained 95% and 97% of their initial efficiencies under 85 °C thermal aging and light soaking in a N_2_ atmosphere for 500 h, respectively (Figure 12c–e) [179].

**Figure 13:**
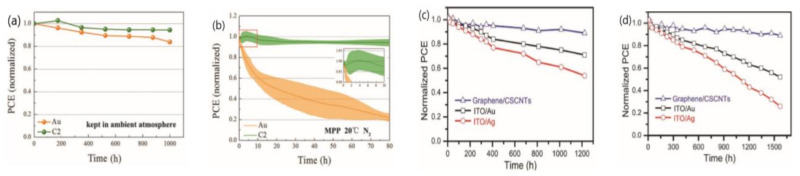
(**a**) Stability test of Au-based devices and self-adhesive microporous carbon (C2)-based devices kept in ambient atmosphere without any encapsulation [184], (**b**) Long-term aging test under constant illumination and MPP in N_2_ atmosphere at 20 °C. The inset shows the detailed degradation characteristics of C2-PSCs during the first 10 h [184]; Efficiency stability of the standard and all-carbon electrode-based flexible PSCs as a function of soaking time under different conditions (**c**) in ambient atmosphere under AM 1.5G illumination in air without a UV filter [180], and (**d**) in ambient atmosphere with constant heating temperature of 60 °C [180].

Replacing the metal electrode with a carbon electrode has been reported to be one of the most effective ways to enhance the device stability of PSCs due to no ion migration, water resistance, and outstanding encapsulation effect. Diverse carbon materials, such as carbon nanotube, carbon fiber, carbon black, graphite, and graphene, have been utilized as counter electrodes. [128,154,180,181] Zhang et al. developed a sort of self-adhesive microporous carbon film as a counter electrode; the PCE reached 19.2%, and the devices exhibited greatly improved long-term stability. More than 95% of the initial efficiency was retained after 1000 h storage under an ambient atmosphere. Furthermore, after aging for 80 h under illumination and maximum power point in a N_2_ atmosphere, the carbon-based PSC retained more than 94% of its initial performance (Figure 13a,b) [128].

Compared with other carbon allotropes, carbon nanotubes (CNTs) are of special interest for the industry because of their high conductivity, excellent mechanical durability, as well as scalable manufacturing capability. Luo et al. fabricated freestanding cross-stacking CNT (CSCNT) films in flexible PSCs. The devices based on CSCNT exhibited good performance preservation, retaining more than 90% of their original efficiencies after 1000 h light soaking or thermal stress in humid air. The improvement of stability was mainly due to the thick and hydrophobic CSCNT layer (≈1.2 µm) combined with Spiro-OMeTAD that provided a stable encapsulating layer to protect perovskite from moisture aggression (Figure 13c,d) [180].

Ni et al. observed light-induced degradation in PSCs that was self-healed completely by resting in the dark for <1 min and was entirely prevented by operating at 0 °C, by which they proposed that the light-stability problem was due to the formation of light-activated meta-stable deep-level trap states [182]. Pulsatile therapy for PSCs has also been developed to prolong device lifetime by addressing the accumulation of both charges and ions in the middle of the maximum power point tracking (MPPT). In Kiwan Jeong’s work, reverse biases were repeatedly applied for a very short time without any pause of operation. Accordingly, the formation of harmful deep-level defects can be prevented, and already formed defects can be cured by driving charge-state transition; this technique is helpful in leading to stabilization of the working device [183].

### 3.5. External Encapsulation Engineering

In the previous achievements, we studied the stability of PSCs from the perspective of the internal material and structure optimization, but in commercial applications, we must also consider the external environment in which the solar cell actually works (such as extreme weather and mechanical damage). The external damage to the devices not only directly affects the operation conditions of the devices but also relates to the environmental problems caused by the decomposition of the materials. Therefore, the external encapsulation of the solar cells is an indispensable segment.

First, we need to strictly select materials that can be applied to the solar cell encapsulation, which at least need to meet the following requirements: (1) High transmittance to incident light, usually higher than 80%, so as to ensure there are enough photons to be absorbed by the perovskite absorber; (2) In order to meet the photovoltaic module 25-year life requirement, the packaging materials need to have stable physical and chemical properties and maintain stability when exposed to extreme environments (such as light soaking, high humidity, high and low temperature); (3) Excellent sealing performance, which requires high water and oxygen permeation resistance to prevent the water and oxygen from diffusing into the interior of functional layers; and (4) Based on the above requirements, the cost of encapsulants should be as low as possible. According to related literature [185,186,187,188,189], the materials currently used in solar cell packaging mainly include some specific resins and polymer films, and related properties are listed in Table 1 and Table 2, respectively.

**Figure 14:**
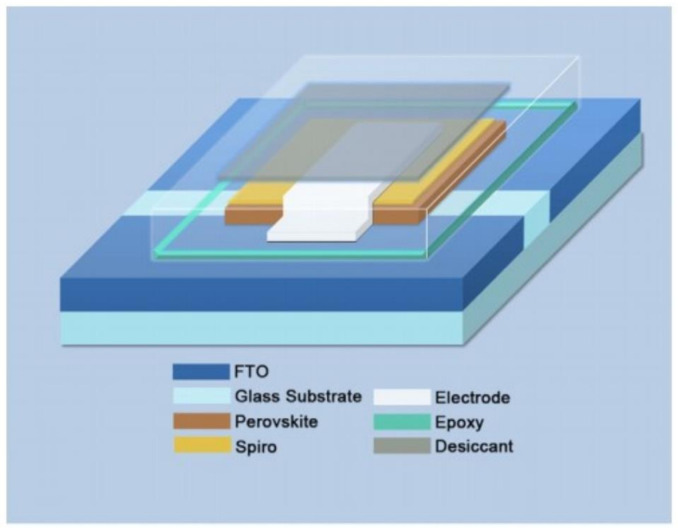
The encapsulation strategies of PSCs [189].

Currently, the most widely used packaging structure is “glass/PSC + sealant/glass” as shown in the Figure 14. [190] First, place the as-prepared solar cell on the bottom glass, then cover the surrounding cell with sealant, and remember to draw forth the electrodes out of the package. After that, cover the top half with another glass. Finally, seal the edges. Much work has been done to demonstrate the validity of this approach, Dong et al [189]. found that encapsulation with UV-curable epoxy resulted in better stability compared to thermally curable epoxy when applied to TiO_2_-based PSCs, which can maintain 85% of the initial efficiency at 85 °C and 65% RH after 144 h. Furthermore, they showed UV-curable epoxy that worked well for TiO_2_-based devices failed to protect ZnO-based devices in rapid degradation tests. However, due to the inherent defects in the light transmittance of resin-based materials, some polymer thin films with better light-transmitting properties have been used to package the PSCs. Fu et al. compared the packaging effect of three polymer materials (PU, POE, and EVA) on small-area solar cells and then transplanted the technique into the package of large-area modules (substrate area 100 cm^2^); their devices maintained 97.52% of the initial efficiency after 2136 h outdoor work [186]. N_2_ filling in the encapsulation process is curial for obtaining long-term PSC stability. Alberti et al. performed encapsulation under a slightly pressurized N_2_ ambient and found that N_2_ not only provided an inert environment, but also played an active role in the stabilization of perovskite surfaces [190].

Cheacharoen et al. designed an optimized packaging technique as shown in the figure; the devices (5 cm × 5 cm) encapsulated with their method passed a 1000-h damp-heat test (85 °C-85%RH) and 200 thermal cycling tests (between −40 °C and 85 °C) [187]. Their work showed that the optimized design of the packaging can well improve the stability of devices.

During the process of commercialization, there is still room to improve the current packaging methods. How to design the electrode extraction mode and ensure the perfect sealing of devices is one of the crucial technical questions of encapsulation. In addition, developing novel packaging materials with higher transmittance and better stability to reduce the loss of efficiency caused by encapsulation also needs our close attention.

In Table 3, we summarize some reports including the test of stability of PSCs in which test conditions significantly differ for different achievements. Except for the aging time and humidity, whether encapsulated, heated or illuminated is inconsistent, which makes it difficult to compare the stability of PSCs between different reports. To sum up, there are several strategies to be introduced to solve stability problems. (1) More alternative materials need to be further developed, such as some inorganic functional materials, which show better stability performance than organic materials. (2) Compositional engineering can be a feasible method to improve device stability. For example, doping of some alkali metal ions can change the tolerance factor of perovskite and make it more stable from the perspective of thermodynamics. (3) Interface engineering is also an important way to protect unstable materials from unfavorable environments or the harmful diffusion of metal atoms and functional groups. Numerous works have been done to achieve extraordinary stability of PSCs, but there is still a long way to go before commercialization.

## 4. Other Issues of Commercialization

In the above sections, we briefly introduced the current progress of PSCs: performance improvement strategies from small-area single cells to large-area modules and stability studies of solar cells from inside to outside (including internal functional layers and external encapsulation). It is concluded that the current laboratory-level PSCs have met the commercial demand in terms of conversion efficiency while stability still needs to be improved, though it can also meet the market requirements through appropriate encapsulation. Therefore, we will further discuss some other issues about commercialization from the perspective of environmental protection and cost-saving, which mainly include Pb toxicity and recycling methods.

### 4.1. Risk and Hazards

As the safety issues once involved in the marketization of CdTe solar cells [199], PSCs also face the same problem because of the existence of Pb in photo-active layers. Researchers have found that CdTe was stable and would hardly cause Cd leakage, while the situation in perovskite was not optimistic. Several factors including moisture and oxygen can cause the degradation of perovskite:

$${\mathrm{MAPbI}}_{3}\overset{H_{2}O}{\Leftrightarrow }{\mathrm{CH}}_{3}{\mathrm{NH}}_{3}I\left(\mathrm{aq}\right)\;+\;{\mathrm{PbI}}_{2}\left(s\right)$$

$${\mathrm{CH}}_{3}{\mathrm{NH}}_{3}I\left(\mathrm{aq}\right)\;↔{\mathrm{CH}}_{3}{\mathrm{NH}}_{2}\left(\mathrm{aq}\right)\;+\;\mathrm{HI}\left(\mathrm{aq}\right)$$

$$2\mathrm{HI}\left(\mathrm{aq}\right)\;\overset{\mathrm{hv}}{\Leftrightarrow }H_{2}+I_{2}\left(s\right)$$

$$4\mathrm{HI}+O_{2}↔2I_{2}+2H_{2}O$$

Therefore, the potential threat of Pb leakage is an inevitable obstacle in the commercialization process. To solve this problem, one of the choices is encapsulation, which has been discussed before. Jiang et al. [200] prepared encapsulated devices with three kinds of packaging materials (UV-cured adhesive, Surlyn resin, and epoxy resin package), then tested their Pb leakage under external conditions. The results showed that the self-healing properties of epoxy resin prevented Pb leakage when the devices were subjected to mechanical damage, high temperature or some other extreme environments; the corresponding Pb leakage rate was as low as 0.08 mg h^−1^ m^−2^.

Although high-quality packaging technology can diminish the risk of Pb leakage into the environment, it must be complemented with a relevant policy to callback the products when reaching their operation lifetime, such as First-Solar Corp. did for their CdTe photovoltaic products. Moreover, encapsulation cannot eliminate the risk as long as the Pb is still there, and because of this, the Pb-free perovskite absorber has attracted great attentions in recent years, including using Ag(I) [201,202], Bi(III) [202,203], Sn(II) [204,205,206,207,208,209], and Ti(IV) [210,211] to replace Pb (II) in perovskite structures. Among these candidates, PSCs based on Sn (II) have the highest power conversion efficiency. The PCE of PSCs based on FA_x_EDA_1−x_SnI_3_ (ethylammonium, EDA) reached 13.24% [207]. As we know, this still lags far behind Pb-based PSCs. In addition, divalent Sn has an intermediate valence, which can be easily oxidized to tetravalent Sn in the air environment. As for other B-position cation perovskites, they are still in the early age, which means they have potential to be applied in solar cells but lack inspiring efficiency breakthrough until now. For example, (CH_3_NH_3_)_3_Bi_2_I_9_-based solar cells had an efficiency of 1.64% in 2017 [203], and CsSn_0.5_Ge_0.5_I_3_ delivered a promising efficiency of 7.11% in 2019 [204]. Even so, these Pb-free perovskite candidates with widely tunable bandgap provide more choices for tandem solar cells.

Briefly speaking, on the one hand, we need to keep upgrading the packaging technology of the PV modules to improve the safety and reliability; on the other hand, the non-Pb-based perovskite materials still need intensive studies so as to be applied in some areas with stringent requirements for safety and environmental protection.

The relatively small amount of Pb contained in PSCs is much more injurious to health caused by the commonly used solvents during the PSCs fabrication process, in contrast to the concerns regarding the water-soluble nature of Pb. [212,213] Solvents including DMF [9,214,215,216], NN-dimethylacetamide [217,218], DMSO [214], and NMP [43,219] are toxic, capable of penetrating skin, and are cancerogenic. Therefore, the development of deposition methods with less hazardous reagents is desired under the background that the PSCs are striving to become largescale commercially viable products. The factors for the transition from hazardous chemicals mainly include (1) cost savings by the shrinkage of expenses and future risks, (2) high-level device efficiency, (3) industry leadership, and (4) advancing corporate stewardship under social responsibility. The potential negative environmental impact could be limited by (1) replacing hazardous reagents with products with improved environment, health, and safety (EHS) properties and/or (2) employing ionic liquids with low vapor pressures, which allows recycling and also eradicates emission to the atmosphere [220,221,222]. Others also tend to use a one-step solution process of perovskites and dedicate to reduce the amount of hazardous solvents, by which the requirement to treat solvents is eliminated [223,224]. Based on these cognitions, it is promising, far-reaching, and worthwhile for the transition to greener chemistry; however, there is difficulty in clarification of which approaches are greener among many methodologies, namely, in classifying and ranking reagents regarding the EHS hazard.

### 4.2. Cost-Related Analysis

**Figure 15:**
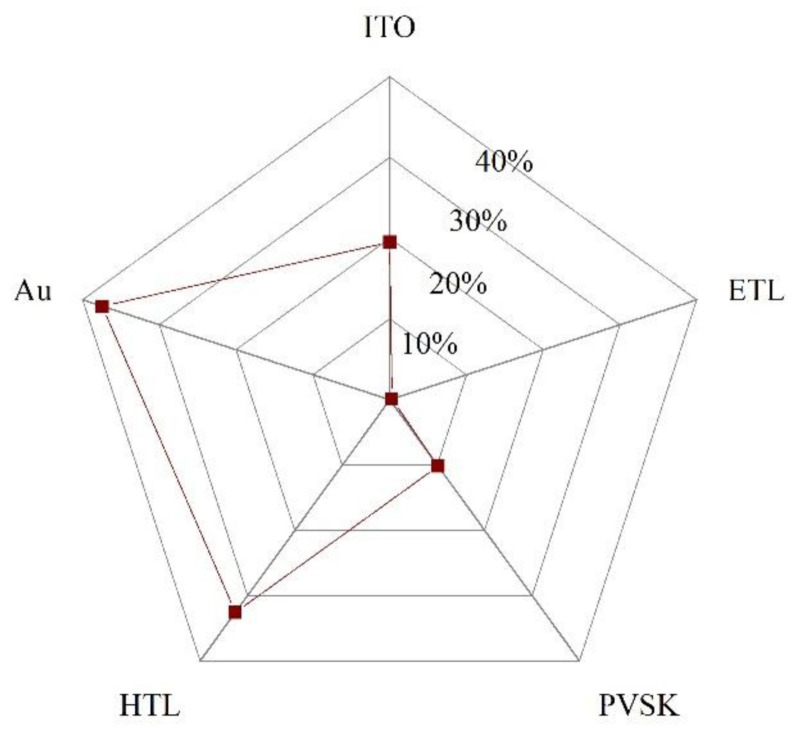
The cost proportion of materials used in laboratory small-area PSCs.

**Figure 16:**
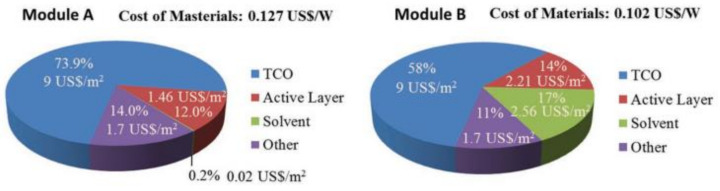
Cost of material distribution for Module A (**left**) and Module B (**right**). The values of materials cost are assumed by the real amount of material used in both the structure and wholesale price. An 80% material usage ratio was considered [225].

Herein, we also evaluated the commercialization prospects of PSCs from the economic perspective. We collected the materials widely used in device preparation, finding that the perovskite material, hole transport layer (mainly Spiro-OMeTAD), and gold electrode occupied a large proportion of the costs. By calculating the raw material cost of the single cell with 2.5 cm × 2.5 cm area prepared in the laboratory, we found that the Spiro and perovskite layers accounted for 65% and 29% of total cost, respectively (Figure 15), according to our laboratory situation. This phenomenon is due to the expensive material price on the one hand, and the spin-coating method does not have the advantage of the thin-film cells causing most of the precursor solution to be wasted in the spin process, which causes the preparation cost to greatly increase on the other hand. Then, we referred to the cost estimation of commercial devices in the literature and found that the large-area preparation methods (including blade-coating, spray-coating, slot-die coating) could effectively reduce the cost of film preparation, and TCO and Au accounted for the main part of the cost at that time (as shown in Figure 16). [225] In our opinion, the cost problems faced by commercialization can be reduced from at least the following aspects: (1) reducing the cost of raw materials; (2) replacing Au electrodes with low-cost carbon electrodes and using cheaper materials as hole transport layers; (3) recycling of transparent conductive films; and (4) efficiency improvement, which is always the best way to reduce the cost of marketization, so optimizing large-area preparation techniques to improve the device conversion efficiency and stability should never stop.

**Figure 17:**
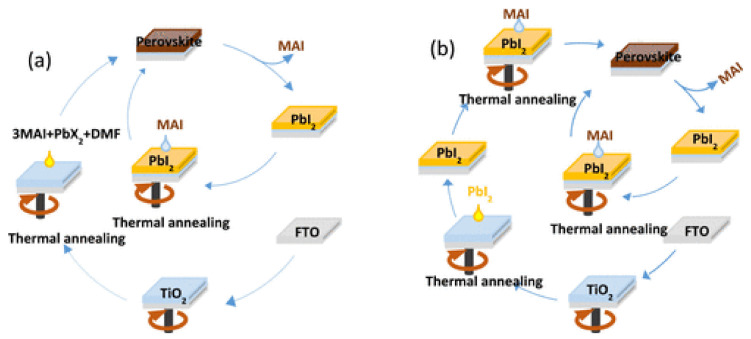
Schematic showing the recycling process for MAPbI_3_ film deposited by (**a**) single-step chloride and single-step acetate route and (**b**) sequential deposition route [228].

For the above points, the reduction of the raw material cost needs the integration of market and technology. Moreover, the study of improved efficiency is always imminent, while the recovery of transparent conductive oxide (TCO) is rarely reported. Compared to other functional layers, the recycling of FTO is more feasible. Because of the high stability of transparent conductive film, we can consider using the “inverse preparation” procedure to dissolve and clean the functional layer on it, and then the FTO can be reused as substrate. Binek et al. reported the procedure to remove every layer of the solar cells separately to recycle the FTO. They peeled off the Au layer by a mechanical method, dissolved the HTL with CB (Chlorobenzene), and then degraded the perovskite layer with water. Finally, the FTO substrate was obtained by washing the residues with DMF [226]. Augustine et al. treated the PSCs with KOH alkaline solution and recycled the ITO for the fabrication of new PSCs; the conversion efficiency of new devices was only 0.85% lower than that of the previous devices [227]. In addition to TCO, the active layer can also be recycled (Figure 17) [228].

However, the encapsulation, energy consumption, and costs of raw materials and the fabrication process must be regarded as actual module production as well. Cai et al. systematically analyzed the total cost of the “humble process” (with moderate efficiency) and “noble process” (with high efficiency) and demonstrated that the costs of these two module structures were lower than those of other PV technologies [225]. The improvement of conversion efficiency and the development of manufacture technology are always the two most effective ways to reduce the cost of PV products, guaranteed by unremitting effort.

Enslaved by the raw material cost, the development of scalable PSCs remains challenging in realizing the low-cost manufacturing of PV systems. Under this background, several cost model studies were done to enhance the knowledge of the production cost, according to the hypothesized device stacks and the production workflows [225,229,230]. Herein, these referred achievements show the cost advantages of PSCs over other PV technologies in a clear manner. Nevertheless, a critical perspective is needed during the judgement of the attempts in evaluating the PSC cost competitiveness since that uncertainty remains in the large-scale implementation of the examined device-stack configurations in the future. This situation indicates the potential innovation opportunities in the materials and/or device-stack configurations for large-scale PSCs and also suggests the hardship in the realistic cost assessments of the deployable PSCs, which is at the initial phase of technology development and is companied by certainty absence related to the module requirements such as encapsulation, weight, and structure. Generally, the most uncertain factor is stability, although continuous progress has been made [230].

## 5. Conclusions and Perspectives

PSCs have experienced rapid progress in the last couple of years and are already treated as a promising candidate for the next generation of photovoltaic technologies. To date, the PCE of the small-area PSC has exceeded 25% [3], while that of the PSM is 11.6% (802 cm^2^) [32]. Even though the performance of small-area PSCs exhibits a dramatic improvement and has reached comparable performance with the conventional Si PV technology, the difficulty in scaling-up and the further improvement of the intrinsic stability have become bottlenecks in PSC industrialization.

In this review, we summarized the achievements in PSCs in recent years. The transition from PSCs to PSMs is described based on the methods of maintaining efficiency during the scaling up process, including scalable coating methods and optimization of the functional layer. In addition, the stability, material cost, and environmental issues must be addressed. There are several key research areas and challenges that need to be expanded upon and addressed for enhancing the commercial potential for perovskite photovoltaic technology. (1) Considerable advances have been achieved in the scalable solution processing for perovskite deposition, exhibiting the coating perovskite films in large-areas (hundreds to thousands of square centimeters) relying on the printing and R2R coating. However, novel film-forming procedures (fabrication method and doping optimization) with developed inks/vapor deposition sources are urgently required to obtain large-scale functional layers with negligible scale-up losses. (2) Designing and fabricating modules is another significant factor to be considered for highly efficient PSMs in the commercialization process. Investigating an advanced scribing technology and reducing the resistance in the interconnection area are essential. (3) Further development of alternative stable materials and continuous innovations on external encapsulation for PSMs are required to achieve device stability and practical application. (4) Negative environmental impacts from functional layers (water-soluble Pb in perovskites) and fabrication procedures (cancerogenic solvents) of PSMs are substantial, thus needing to be carefully managed for PSM industrial adoption. (5) Cost analysis is another important aspect that needs to be carefully evaluated for commercialization of PSMs. Herein, the development of mass fabrication techniques, with production capability comparable to the commercially available ones, such as Si, CIGS, and CdTe, is crucial to advance cost-competitive perovskite-based tandem solar cells.

## Figures and Tables

**Table 1:** Properties of commercial epoxy used in solar cell encapsulation.

Materials	Provider	Curing Condition	Appearance	Hardness (D)
UV-curable epoxy	ThreeBond	60 kJ/m^2^, 80 °C, 1 h	pinkish white	76
AB epoxy glue	Super Glue Corp.	25 °C, 4–6 min	Hazy white	75–85
Thermally curable epoxy	Kyoritsu Chemical	6000 mJ/cm^2^, 80 °C, 30 min	——	83

**Table 2:** Properties of commercial encapsulants used in solar cell encapsulation.

Materials	Provider	Transmittance (%)	Elastic Modulus (MPa)	Possibly Harmful
EVA	Mitsui Chemicals	93	10	Acetic acid
Surlyn	Dupont	93.4	397	Methacrylic acid
Polyolefin	3M	91	9.1	Unknown

**Table 3:** Stability of PSCs.

Perovskite Materials	Device Structure	Active Area/mm^2^	Initial PCE (%)	Test Condition	Stability Result	Published Year	Ref.
Cs_0.05_(FA_0.92_MA_0.08_)_0.95_ Pb(I_0.92_Br_0.08_)_3_	ITO/PTAA/PVSK /C_60_/BCP/Cu	6.69	23	MMP tracking on encapsulated devices under AM1.5 illumination for 1000 h	Negligible decrease	2020	[191]
				T = 85 °C, for 1020 h in N_2_ environment	lost around 10%		
Cs_0.1_MA_0.2_FA_0.7_Pb_0.5_Sn_0.5_I_3_	ITO/ PEDOT:PSS/PVSK/PCBM/ PEIE/Ag	4.9	18.95	Under full AM1.5G illumination (without UV-filter) after 8.6 h	lost around 10%	2020	[192]
(K_x_(Cs_0.05_(FA_0.85_MA_0.15_)_0.95_Pb(I_0.85_Br_0.15_)_3_	FTO/SnO_2_/PVSK/Spiro-OMeTAD/Au	16	20.56	Unencapsulated devices stored under ambient air conditions with 10 ± 5 RH% over 1000 h	no decrease	2017	[160]
FA_0.75_Cs_0.25_Sn_0.5_Pb_0.5_I_3_	ITO/ PEDOT:PSS/PVSK/C_60_/BCP/Ag		15.6	Under continuous one-sun illumination over 30 h	no decrease	2018	[193]
Rb-FA_0.75_MA_0.15_Cs_0.1_PbI_2_Br	FTO/TiO_2_/PVSK/PTAA/Au	17.64	17.4	under continuous one-Sun illumination after >12 h under N_2_ environment and the T = 25 °C	lost around 5%	2017	[159]
Rb- Cs_0.05_(MA_0.17_FA_0.83_)_0.95_Pb(I_0.83_Br_0.17_)_3_	ITO/TiO_2_/PVSK/PTAA/Au	4	18.03	Unencapsulated devices stored at 85 °C, under dry air (<5% humidity), after 347 h	lost around 26%	2018	[194]
(FA_0.65_MA_0.20_Cs_0.15_)Pb(I_0.8_Br_0.2_)_3_	ITO/PTAA/PVSK/C_60_/ BCP/Ag	6	19.8	Stored at room temperature under N_2_ environment for over 4000 h	lost around 4%	2019	[195]
(FA_0.65_MA_0.2_Cs_0.15_)Pb(I_0.8_Br_0.2_)_3_	ITO/PTAA/PVSK/C_60_/ BCP/Ag		20.7	Unencapsulated devices tested under continuous illumination in an N_2_-filled environment for 1000 h	lost around 20%	2020	[80]
MAPbI_3−x_(SeCN)_x_	FTO/TiO_2_/PVSK/Spiro-oMeTAD/Ag	16	18.41	Placed in N_2_-filled glove box after continuous testing for 500 h (tested every 24 h)	lost around 14%	2019	[196]
(CsPbI_3_)_0.04_ (FAPbI_3_)_0.82_ (MAPbBr_3_)_0.14_	FTO/SnO_2_/PVSK/Spiro-OMeTAD/Au	9	21.38	Test at room temperature and 70% humidity and stored in ≈25% humidity at RT in the dark over 1080 h without encapsulation	lost around 10%	2020	[126]
Cs_0.05_(MA_0.17_FA_0.83_)_0.95_Pb(I_0.83_Br_0.17_)_3_	FTO/TiO_2_/PVSK/Spiro-OMeTAD/Au	12	21.6	Encapsulated devices under continuous one sun illumination (≈30 °C, ≈35% humidity) for 300 h	lost around 24%	2019	[197]
(FAPbI_3_)_0.85_ (MAPbBr_3_)_0.15_	ITO/NiO_x_/PVSK/PCBM/ZrAcac/Ag	7.5	20.67	Encapsulated devices under continuous one-sun illumination (≈23 °C, ≈35% humidity) for 200 h	lost around 24%	2019	[127]
(FAPbI_3_)_0.8_ (MAPbBr_3_)_0.2_	ITO/ PEDOT:PSS/PVSK/PCBM/ ZnO/Ag	13.5	20.3	unencapsulated devices tested under continuous one sun illumination (25 °C, 60% humidity) over 170 h	lost around 16%	2018	[171]
MAPb_1−x_Mn_x_I_3_	FTO/TiO_2_/PVSK/Spiro-OMeTAD/Ag	9	19.09	unencapsulated devices stored in an ambient atmosphere (30% relative humidity and 25 °C) for 48 h	lost around 5%	2019	[191]
Rb_5_Cs_10_FAPbI_3_	FTO/SnO_2_/PCBM: PMMA/PVSK/PMMA/Spiro-OMeTAD/Au	10.24	20.35	1000 h of continuous MMP tracking under N_2_ atmosphere at room temperature	lost around 2%	2018	[158]
(FA_x_MA_1−x_)Pb(I_y_Cl_1-y_)_3_	ITO/SnO_2_/PVSK/Spiro-OMeTAD/Au	9	22.03	unencapsulated devices stored in the atmosphere at 66% humidity after 110 h	lost around 22%	2020	[198]
(FAPbI_3_)_1−x_(MAPbBr_3_)_x_	FTO/TiO_2_/PVSK/Spiro-OMeTAD/Au		19.3	Stored at 25 °C with the humidity of 40% after 700 h, in dark	lost around 3%	2019	[114]
MAPb(I_x_Br_1−x_)_3_	ITO/ PEDOT:GO/PVSK/PCBM/ ZnO/Ag	13.5	18.09	encapsulated devices were measured under an ambient air condition after 25 days	lost around 20%	2018	[107]
MAPbI3−xClx	ITO/ PEDOT:PSS/PVSK/PCBM/ RhB101/Ag/LiF	11	18.0	unencapsulated devices were stored in ambient environment with 55 ± 5% humidity after 240 h	lost around 50%	2018	[105]
MAPbI_x_Cl_3−x_	ITO/PEDOT:SAF/PVSK/PCBM/BCP/Ag	12.5	16.2	unencapsulated devices were stored in ambient air with RH% ∼30% for 44 days without excluding the room light	lost around 10%	2019	[113]
MAPb(I_0.9_Br_0.1_)_3_	FTO/NiO:5K/PVSK /PCBM/C60/BCP/Ag		18.05	unencapsulated devices were stored in ambient air with RH% 15 ± 5% for 240 h	lost around 10%	2019	[140]

## Data Availability

The study does not include publicly archived datasets.

## Supplementary Materials

The following are available online at https://www.mdpi.com/article/10.3390/ma14216569/s1, Table S1: Reports on perovskite solar modules from 2014 to July 2021.
