# Supplementary material for: Progress in Perovskite Solar Cells towards Commercialization—A Review
Source: Materials (Basel). 2021 Nov 1;14(21):6569. doi: 10.3390/ma14216569 (PMC8585319; doi:10.3390/ma14216569)
Supplement: Supplementary file 1 [file materials-14-06569-s001.zip › materials-1381352.pdf]

materials

MDPI

Supplementary Materials

Progress in Perovskite Solar Cells towards Commercialization—A Review

Hongqiao Wang [1], Yunfan Wang [2], Zhipeng Xuan [1], Tingting Chen [1], Jingquan Zhang [1,3], Xia Hao [1,3,*], Lili Wu [1,3], Iordania Constantinou [4,5] and Dewei Zhao [1,3]

[1] College of Materials Science and Engineering & Institute of New Energy and Low-Carbon Technology, Sichuan University, Chengdu 610065, China; 2019223010007@stu.scu.edu.cn (H.W.); 2018226220003@stu.scu.edu.cn (Z.X.); chentingting1@stu.scu.edu.cn (T.C.); zhangjq@scu.edu.cn (J.Z.); wulili@scu.edu.cn (L.W.); dewei.zhao@scu.edu.cn (D.Z.)
[2] Department of Materials Science and Engineering, City University of Hong Kong, Kowloon Tong, Hong Kong 999077, China; yunfawang2-c@my.cityu.edu.hk
[3] Engineering Research Center of Alternative Energy Materials & Devices, Ministry of Education, Chengdu 610065, China
[4] Institute of Microtechnology (IMT), Technische Universität Braunschweig, Alte Salzdahlumer Str. 203, 38124 Braunschweig, Germany; i.constantinou@tu-braunschweig.de
[5] Center of Pharmaceutical Engineering (PVZ), Technische Universität Braunschweig, Franz-Liszt-Str. 35a, 38106 Braunschweig, Germany
* Correspondence: hao.xia0808@scu.edu.cn

Citation: Wang, H.; Wang, Y.; Xuan, Z.; Chen, T.; Zhang, J.; Hao, X.; Wu, L.; Constantinou, I.; Zhao, D. Progress in Perovskite Solar Cells Towards Commercialization—A review. *Materials* **2021**, *14*, 6569. https://doi.org/10.3390/ma14216569

Academic Editor: Fabrice Goubard

Received: 30 August 2021
Accepted: 26 October 2021
Published: 1 November 2021

Publisher's Note: MDPI stays neutral with regard to jurisdictional claims in published maps and institutional affiliations.

Copyright: © 2021 by the authors. Licensee MDPI, Basel, Switzerland. This article is an open access article distributed under the terms and conditions of the Creative Commons Attribution (CC BY) license (http://creativecommons.org/licenses/by/4.0/).

Table S1. Reports on perovskite solar modules (PSMs) from 2014 to July 2021.

Pub. Date	Module structure	Methods	GFF (%)	Active/Aperture area (cm[2])	Active/Aperture PCE (%)	Flexible (Yes / No)	Ref.
2014.12.05	FTO/TiO_2/$MAPbCl_3$/CuPc/Au	Spin-coating	*	*/8	*/2.66	No	[1]
2014.12.24	FTO/TiO_2/$MAPbI_3$/Spiro-OMeTAD/Au	Blade-coating	73	10.1/13.83	10.4/7.59	No	[2]
2015.07.24	FTO/NR-TiO_2/$MAPbCl_3$/Spiro-OMeTAD/Au	Screen printing	*	10.8/*	10.5/*	No	[3]
2016.01.05	ITO/TiO_2/$MAPbI_xCl_{3-x}$/Spiro-OMeTAD/Au	Spin-coating	91	3.64/4	14.9/13.6	No	[4]
2016.07.08	FTO/TiO_2/$MAPbI_3$/Spiro-OMeTAD/Au	Chemical vapor deposition (CVD)	*	8.8/~18.5	9.5/~4.5	No	[5]
2016.07.10	ITO/NiO/$MAPbI_3$/PCBM/ZnO/TCA/AgNWs	Blade-coating	91	*/3.3	*/9.75	Yes	[6]
2016.07.30	ITO/PEDOT:PSS/$MAPbI_xCl_yBr_{3-x-y}$/PCBM/Al	Spin-coatign	*	25.2/*	14.3/*	No	[7]
206.09.20	FTO/TiO_2/$MAPbI_xCl_{3-x}$/PTAA/Au	Spray-coating	40	40/100	15.5/6.2	xx	[8]
2016.10.20	FTO/NiO/$MAPbI_3$/PCBM/Ag	Spin-coating	60	15/25	12/7.2	No	[9]
2016.10.27	FTO/TiO_2-Graphee/GO-Li/$MAPbI_3$/Spiro-OMeTAD/Au	Spin-coating	73	50.56/69.52	12.6/9.20	No	[10]
2016.11.09	FTO/TiO_2/ZrO_2/$MAPbI_3$/Carbon	Screen printing	70	70/100	10.74/7.52	No	[11]
2017.01.31	FTO/TiO_2/ZrO_2/$MAPbI_3$/Carbon	Screen printing	96	49/51	10.4/9.98	No	[12]
2017.02.17	ITO/PEDOT:PSS/$MAPbI_3$/PCBM/Al	Spin-coating	*	11.25/*	15.4/*	No	[13]
2017.05.20	FTO/TiO_2/$MAPbI_xCl_{3-x}$/Spiro-OMeTAD/Au	Blade-coating	88	11.09/12.6	14.06/12.37	No	[14]
2017.06.24	ITO/NiO/$MAPbI_3$/PCBM-BCP/Ag	Spin-coating	54	13.5/25	11.8/6.37	No	[15]
2017.07.18	FTO/TiO_2/$MAPbI_3$/Spiro-OMeTAD/Au	Spin-coating	57	14.3/25	14.28/8.17	No	[16]
2017.02.17	FTO/TiO_2/$MAPbI_3$/Spiro-OMeTAD/Au	Pressure-assisted processing	*	*/36	*/12.1	No	[17]
2017.09.07	FTO/TiO_2/$MAPbI_3$/Spiro-OMeTAD/Au	Spin-coating	95	14.5/15.28	9.3/8.37	No	[18]
2017.10.05	FTO/TiO_2-SnO_2/$MAFAPbI_xBr_{3-x}$/Spiro-OMeTAD/Au	Spin-coating	*	10/*	14.39/*	No	[19]
2017.11.16	ITO/TiO_2/$MAPbI_xCl_{3-x}$/Spiro-OMeTAD/Au	Slot die coating	95	142/149.5	11.8/11.2	No	[20]
2017.11.22	ITO/TiO_2/$CsFAPbI_3$/Spiro-OMeTAD/Au	CVD	*	12/*	14.6/*	No	[21]
2017.11.28	FTO/TiO_2/$MAPbI_3$/Spiro-OMeTAD/Au	Spin-coating	*	10.1/*	13/*	No	[22]
2018.01.02	FTO/TiO_2/$MAFAPbI_3$/Spiro-OMeTAD/Au	Blade-coating	87	9.01/10.36	17.9/15.6	No	[23]

2018.01.11	ITO/C_{60}/$MAPbI_3$/ Spiro-OMeTAD/MoO_3-Au	Spin-coating	*	16/*	12.7/*	Yes	[24]
2018.03.14	FTO/TiO_2-SnO_2/$MAFAPbI_3$/Spiro-OMeTAD/Au	Chemical deposition	*	10.56/*	16.31/*	No	[25]
2018.04.10	FTO/SnO_2/$KCsFAMAPbI_xBr_{3-x}$/Spiro-OMeTAD/Au	Spin-coating		*/10	*/12.4	Yes	[26]
2018.04.30	FTO/SAH-TiO_2/$MAPbI_3$/Spiro-OMeTAD/Au	Soft-Cover-Assisted deposition	*	*/16	*/14.01	No	[27]
2018.05.12	FTO/TiO_2-SnO_2/$MAPbI_3$/Spiro-OMeTAD/Au	Spin-coating	*	12/*	8.8/*	Yes	[28]
2018.05.14	ITO/PTAA/$MAPbI_3$/C_{60}/BCP/Cu	Blade-coating	93	*/33<br>*/57.2	*/15.3<br>*/14.6	No	[29]
2018.08.13	FTO/TiO_2/ZrO_2/$MAPbI_3$/Carbon	Screen printing	46	198/435.6	6.6/3.03	No	[30]
2018.09.01	FTO/SnO_2/$MAPbI_3$/Spiro-OMeTAD/Au	Spin-coating	64	16/25	11.5/7.36	No	[31]
2018.10.15	PDMS/PEDT:PSS/$FAMAPbI_xBr_{3-x}$/PCBM/PEI-PEDOT:PSS/PDMS	Spin-coating	*	*/56.02	*/7.91	Yes	[32]
2018.11.02	ITO/SnO_2/$CsFAMAPbI_3Br_{3-x}$/Spiro-OMeTAD/Au	Slot-die printing	*	*/16.07	*/15.22	Yes	[33]
2018.11.02	FTO/SnO_2/2D-3D perovskite/Spiro-OMeTAD/Au	Printing method	*	57/*	11.59/*	No	[34]
2019.02.14	FTO/SnO_2/$FAPbI_xBr_{3-x}$/Spiro-OMeTAD/Au	CVD	*	12/*	14.7/*	No	[35]
2019.02.18	FTO/SnO_2/C_{60}/$CsFAPbI_xBr_{3-x}$/Spiro-OMeTAD/Au	CVD	90	82.6/91.8	10.37/9.34	No	[36]
2019.02.27	FTO/TiO_2/ZrO_2/$MAPbI_3$/Cu:NiO_x/Carbon	Screen printing	70	70/100	12.1/8.47	No	[37]
2019.03.15	FTO/SnO_2/$FAMAPbI_xBr_{3-x}$/Spiro-OMeTAD/Au	Spin-coating	*	*/53.64	*/13.85	No	[38]
2019.03.27	FTO/TiO_2/$FAMAPbI_xBr_{3-x}$/WBH/P3HT/Au	Bar-coating	*	*/24.97	*/17.1	No	[39]
2019.04.15	FTO/TiO_2-SnO_2/$FAMAPbI_xBr_{3-x}$/Spiro-OMeTAD/Au	Spin-coating	*	*/15	*/16.73	No	[40]
2019.04.29	FTO/TiO_2/$MAPbI_3$/Spiro-OMeTAD/Au	Blade-coating	81	22/27.25	14.2/11.5	No	[41]
2019.05.16	ITO/CuO/$MAPbI_xBr_{3-x}$/PCBM/BCP/Ag	Spin-coating	93	25/26.88	15/14	No	[42]
2019.06.25	FTO/TiO_2/$MAPbI_3$/Spiro-OMeTAD/Au	Blade-coating	91<br>87	14.2/15.91<br>47.68/55	12.85/11.7<br>14.7/12.7	No	[43]
2019.07.03	PET/PEDOT:PSS/$FAMAPbI_xBr_{3-x}$/PCBM/Ag	Slot-die printing	*	*/25	*/10.9	Yes	[44]
2019.07.04	FTO/SnO_2/$FAMAPbI_xBr_{3-x}$/Spiro-OMeTAD/Au	Spin-coating	93	*/53.64	*/17.4	No	[45]
2019.09.16	FTO/TiO_2/$MAPbI_3$/Carbon	Printing method	*	52/*	10.2/*	No	[46]
2019.11.21	ITO/PTAA/$MAPbI_3$/C_{60}/BCP/Cu	Blade-coating	*	*/42.9	*/15.86	Yes	[47]
2019.12.13	FTO/SnO_2/$MAPbI_3$/Spiro-OMeTAD/Au	Sputtered	91	20.75/22.8	13.22/12.03	No	[48]

2019.12.16	FTO/TiO_2/$MAPbI_xCl_{3-x}$/Spiro-OMeTAD/Au	Spin-coating	96	39.17/40.8	9.41/9.04	No	[49]
2020.01,03	FTO/TiO_2/$CsFAMAPbI_xBr_{3-x}$/Spiro-OMeTAD/Au	Blade-coating	*	50/*<br>17/*	11.6/*<br>12.7*	No	[50]
2020.01.15	FTO/NiO_x/$CsPbI_xBr_{3-x}$/ZnO-C_{60}/Ag	Quasi-curved heating method	*	10.92/*	12/*	No	[51]
2020.01.15	ITO/PEDOT:EVA/$FAMAPbI_xBr_{3-x}$/PCBM/Ag	Scribing method	87	31.2/35.86	17.55/15.21	Yes	[52]
2020.01.17	FTO/SnO_2/$CsFAMAPbI_xBr_{3-x}$/Spiro-OMeTAD/Au	spin-coating	91	*/25.49	*/18.73	No	[53]
2020.01.29	FTO/SnO_2/$MAPbI_3$/Spiro-OMeTAD/Au	Spray-coating	87	15/17.24	9.37/8.15	No	[54]
2020.02.14	FTO/TiO_2/ZrO_2/$MAPbI_3$/Carbon-Cu	Screen printing	*	11.7/*	11/*	No	[55]
2020.03.11	FTO/ZnO-ZnS/TiO_2/$MAPbI_3$/Spiro-OMeTAD/Au	Dip-coating	*	*/12	*/16	No	[56]
2020.04.01	FTO/TiO_2/ZrO_2/$MAPbI_3$/Carbon	Scribing method	*	*	10.37/*	No	[57]
2020.04.02	FTO/SnO_2/PCBM/$MAPbI_3$/Spiro-OMeTAD/Au	Co-evaporated	72	21/29.16	18.13/13.05	No	[58]
2020.04.07	FTO/SnO_2/$FAMAPbI_xBr_{3-x}$/Spiro-OMeTAD/Au	Blade-coating		*/10<br>*/53.6	*/16.54<br>*/13.32	No	[59]
2020.04.16	FTO/SnO_2/$CsFAMAPbI_xBr_{3-x}$/Spiro-OMeTAD/Au	D-bar coating	75	18.66/25	*/17.01	No	[60]
2020.04.24	FTO/ZnO-ZnS/TiO_2/$CsPbI_3$/Spiro-OMeTAD/Au	Blade-coating	*	*/16	*/11.8	No	[61]
2020.04.28	FTO/TiO_2/ZrO_2/$MAPbI_3$/Carbon	Slot-die coating	75	60.08/80.55	12.87/9.65	No	[62]
2020.05.07	FTO/ PEDOT:PSS/$MAPbI_3$/C_{60}/BCP/Ag	Blade-coating	*	10.56/*	13.03/*	No	[63]
2020.05.11	FTO/SnO_2/$FAMAPbI_3Br_{3-x}$/Spiro-OMeTAD/Au	Spin-coating	98	24.5/25	16.27/16	No	[64]
2020,06.25	FTO/SnO_2/$FAMAPbI_3$/Spiro-OMeTAD/Au	Spin-coating		*/25.9	*/17.33	No	[65]
2020.08.29	ITO/PEDOT:PSS/2T-NATA/$MAPbI_3$/C_{60}/BCP/ Ag	Evaporated	*	16/*	15.06/*<br>13.15/*	Yes	[66]
2020.10.19	FTO/SnO_2/$FACsPbI_3$/Spiro-OMeTAD/Au	Chemical vapor deposition	90	*/22.4	*/12.3%	No	[67]
2020.10.20	FTO/TiO_2/$FAMAPbI_xCl_{3-x}$/$[M_4N]BF_4$/Spiro-OMeTAD/Au	Slot-die coating	86	10.2/11.86<br>7.92/9.2	18.6/15.9<br>19.6/16.77	No	[68]
2020.10.22	FTO/Zn_2SnO_4/SnO_2/$FACsPbI_3$/Spiro-OMeTAD/Au	Blade-coating	90	*/100<br>*/225<br>*/400	*/15.5<br>*/12.9<br>*/11.8	Yes	[69]
2020.12.09	FTO/TiO_2/Perovskite/PTAA/Au	Spin-coating	86	42.8/50	17.05/14.66	No	[70]
2020.12.16	ITO/NiO/$CsFAPbI_3$/C_{60}/BCP/Ag	Spray-coating	90	5.9/6.55	15.2/13.68	No	[71]
2020.12.17	FTO/TiO_2/$CsFAPbI_3$/Spiro-OMeTAD/Au	Spin-coating	*	7.2/*	16.3/*	No	[72]

2020.12.18	FTO/SnO_2/$FAMAPbI_xBr_{3-x}$/Spiro-OMeTAD/Au	D-bar coating		19.69/*	17.46/*	No	[73]
2020.12.21	FTO/ZnO-ZnS/$FACsPbI_3$/Spiro-OMeTAD/Au	Spin-coating	*	49/*	13.84/*	No	[74]
2020.12.21	FTO/TiO_2/SnO_2/$FACsPbI_xCl_{3-x}$/Spiro-OMeTAD /Au	Slot-die coating	*	*/35.8	*/15.3	No	[75]
2021.01.24	PEN/PEDOT:PSS/Perovskite/PCBM/Ag	Meniscus-coating	*	15/*	16.15/*	Yes	[76]
2021.01.25	FTO/SnO_2/$FAMAPbI_xCl_{3-x}$/Spiro-OMeTAD/Au	Spin-coating	84	77.47/91.8	12.14/10.25	No	[77]
2021.03.02	FTO/TiO_2/GO-K/$FACsPbI_xBr_{3-x}$/Spiro-OMeTAD /Au	Spin-coating	88	16/18.18	16.1/14.17	No	[78]
2021.03.05	PET/SnO_2/$FAMACsPbI_xBr_{3-x}$/Spiro-OMeTAD/Au	Spin-coating	87	21.8/25.05	11.7/10.18	No	[79]
2021.03.15	ITO/SAM/ $FAMACsPbI_xBr_{3-x}$/LiF/C_{60}/SnO_2/Cu	Spin-coating	*	19.4/*	2.2/*	No	[80]
2021.03.21	ITO/PEDOT:PSS/$MAPbI_3$/PCBM/BCP/Ag	Spin-coating	70	10/14.28	8.8	No	[81]
2021.03.29	ITO/PTAA/$MAPbI_xCl_{3-x}$/C_{60}/BCP/Cu	Blade-coating	*	*/60.8	*/16.3	No	[82]
2021.04.30	ITO/NiMgLiO/$FACsPbI_xBr_{3-x}$/LiF/C_{60}/BCP/Bi/ Ag	Slot-die coating	93	19.29/20.77	/15.42	No	[83]
2021.05.03	ITO/PTAA/$MAFAPbI_3$/C_{60}/BCP/Cu	Blade-coating	94	*/18.2	*/35.8	No	[84]
2021.05.07	ITO/SnO_2/$FAPbI_{3-x}Br_x$/Spiro-OMeTAD/Au	Spin-coating	89	19.93/22.4	16.01/14.22	No	[85]
2021.06.09	ITO/SnO_2/$MAPbI_3$/P3HT/Ta-WO_x/Carbon	Blade-coating	*	*/*	15.3	No	[86]
2021.06.16	PET/PTAA/ $FACsPbI_xBr_{3-x}$/C_{60}/BCP/Ag	Blade-coating		15.7/*	10.51/*	Yes	[87]
2021.07.08	FTO/PTAA/$FACsPbI_3$/Spiro-OMeTAD/Au	Spin-coating	*	18/*	18.1/*	No	[88]
2021.07.09	FTO/TiO_2/ $FAPbI_3$/Spiro-OMeTAD /Au	Spin-coating	90	23.27/25.74	20.75/19.76	No	[89]
2021.07.10	FTO/SnO_2/$FAMACsPbI_{3-x}Br_x$/Spiro-OMeTAD/Au	Spin-coating	91<br>86	22.4/24.75<br>91.8/106.74	17.26/15.62<br>13.72/11.79	No	[90]
2021.07.20	ITO/SnO_2/EDTAK/$FAMACsPbI_{3-x}Br_x$/Spiro-OMeTAD/Au	Spin-coating	*	*/22.4	*/16.6	No	[91]
2021.07.21	FTO/NiO_x/PTAA/Al_2O_3/Perovskite/PCBM/BCP/SnO_2/Ag	Spin-coating	*	11.2/*	16.9/*	No	[92]

Note: In this table, related parameters of PSMs reported from 2014 to 2021 are presented, reflecting the power conversion efficiency (PCE) and module area development of PSMs.

References

1. Kumar, C.V.; Sfyri, G.; Raptis, D.; Stathatos, E.; Lianos, P., Perovskite solar cell with low cost Cu-phthalocyanine as hole transporting material. *RSC Advances* **2015,** *5* (5), 3786-3791.
2. Razza, S.; Di Giacomo, F.; Matteocci, F.; Cina, L.; Palma, A.L.; Casaluci, S.; Cameron, P.; D'epifanio, A.; Licoccia, S.; Reale, A., Perovskite solar cells and large area modules (100 cm2) based on an air flow-assisted PbI2 blade coating deposition process. *Journal of Power Sources* **2015**, *277*, 286–291.
3. Fakharuddin, A.; Di Giacomo, F.; Palma, A.L.; Matteocci, F.; Ahmed, I.; Razza, S.; D'Epifanio, A.; Licoccia, S.; Ismail, J.; Di Carlo, A., Vertical TiO2 nanorods as a medium for stable and high-efficiency perovskite solar modules. *ACS nano* **2015,** *9* (8), 8420-8429.

4. Qiu, W.; Merckx, T.; Jaysankar, M.; De La Huerta, C.M.; Rakocevic, L.; Zhang, W.; Paetzold, U.; Gehlhaar, R.; Froyen, L.; Poortmans, J., Pinhole-free perovskite films for efficient solar modules. *Energy & Environmental Science* **2016,** *9* (2), 484-489.
5. Kim, K.-S.; Lee, H.-J.; Lee, C.; Lee, S.-K.; Jang, H.; Ahn, J.-H.; Kim, J.-H.; Lee, H.-J., Chemical vapor deposition-grown graphene: The thinnest solid lubricant. *ACS nano* **2011,** *5* (6), 5107-5114.
6. Spyropoulos, G.D.; Quiroz, C.O.R.; Salvador, M.; Hou, Y.; Gasparini, N.; Schweizer, P.; Adams, J.; Kubis, P.; Li, N.; Spiecker, E., Organic and perovskite solar modules innovated by adhesive top electrode and depth-resolved laser patterning. *Energy & Environmental Science* **2016,** *9* (7), 2302-2313.
7. Chiang, C.-H.; Lin, J.-W.; Wu, C.-G., One-step fabrication of a mixed-halide perovskite film for a high-efficiency inverted solar cell and module. *Journal of Materials Chemistry A* **2016,** *4* (35), 13525-13533.
8. Heo, J.H.; Lee, M.H.; Jang, M.H.; Im, S.H., Highly efficient CH 3 NH 3 PbI 3− x Cl x mixed halide perovskite solar cells prepared by re-dissolution and crystal grain growth via spray coating. *Journal of Materials Chemistry A* **2016,** *4* (45), 17636-17642.
9. Liao, H.C.; Guo, P.; Hsu, C.P.; Lin, M.; Wang, B.; Zeng, L.; Huang, W.; Soe, C.M.M.; Su, W.F.; Bedzyk, M.J., Enhanced efficiency of hot-cast large-area planar perovskite solar cells/modules having controlled chloride incorporation. *Advanced Energy Materials* **2017,** *7* (8), 1601660.
10. Agresti, A.; Pescetelli, S.; Palma, A.L.; Del Rio Castillo, A.E.; Konios, D.; Kakavelakis, G.; Razza, S.; Cinà, L.; Kymakis, E.; Bonaccorso, F., Graphene interface engineering for perovskite solar modules: 12.6% power conversion efficiency over 50 cm2 active area. *ACS Energy Letters* **2017,** *2* (1), 279-287.
11. Priyadarshi, A.; Haur, L.J.; Murray, P.; Fu, D.; Kulkarni, S.; Xing, G.; Sum, T.C.; Mathews, N.; Mhaisalkar, S.G., A large area (70 cm 2) monolithic perovskite solar module with a high efficiency and stability. *Energy & Environmental Science* **2016,** *9* (12), 3687-3692.
12. Hu, Y.; Si, S.; Mei, A.; Rong, Y.; Liu, H.; Li, X.; Han, H., Stable large-area (10× 10 cm2) printable mesoscopic perovskite module exceeding 10% efficiency. *Solar Rrl* **2017,** *1* (2), 1600019.
13. Chiang, C.-H.; Nazeeruddin, M.K.; Grätzel, M.; Wu, C.-G., The synergistic effect of H 2 O and DMF towards stable and 20% efficiency inverted perovskite solar cells. *Energy & Environmental Science* **2017,** *10* (3), 808-817.
14. Yang, M.; Li, Z.; Reese, M.O.; Reid, O.G.; Kim, D.H.; Siol, S.; Klein, T.R.; Yan, Y.; Berry, J.J.; Van Hest, M.F., Perovskite ink with wide processing window for scalable high-efficiency solar cells. *Nature Energy* **2017,** *2* (5), 1-9.
15. Troughton, J.; Hooper, K.; Watson, T.M., Humidity resistant fabrication of CH3NH3PbI3 perovskite solar cells and modules. *Nano Energy* **2017**, *39*, 60–68.
16. Bu, T.; Wu, L.; Liu, X.; Yang, X.; Zhou, P.; Yu, X.; Qin, T.; Shi, J.; Wang, S.; Li, S., Synergic interface optimization with green solvent engineering in mixed perovskite solar cells. *Advanced Energy Materials* **2017,** *7* (20), 1700576.
17. Chen, H.; Ye, F.; Tang, W.; He, J.; Yin, M.; Wang, Y.; Xie, F.; Bi, E.; Yang, X.; Grätzel, M., A solvent-and vacuum-free route to large-area perovskite films for efficient solar modules. *Nature* **2017,** *550* (7674), 92-95.
18. Palma, A.L.; Matteocci, F.; Agresti, A.; Pescetelli, S.; Calabrò, E.; Vesce, L.; Christiansen, S.; Schmidt, M.; Di Carlo, A., Laser-patterning engineering for perovskite solar modules with 95% aperture ratio. *IEEE Journal of Photovoltaics* **2017,** *7* (6), 1674-1680.
19. Liu, X.; Bu, T.; Li, J.; He, J.; Li, T.; Zhang, J.; Li, W.; Ku, Z.; Peng, Y.; Huang, F., Stacking n-type layers: Effective route towards stable, efficient and hysteresis-free planar perovskite solar cells. *Nano Energy* **2018**, *44*, 34–42.
20. Di Giacomo, F.; Shanmugam, S.; Fledderus, H.; Bruijnaers, B.J.; Verhees, W.J.; Dorenkamper, M.S.; Veenstra, S.C.; Qiu, W.; Gehlhaar, R.; Merckx, T., Up-scalable sheet-to-sheet production of high efficiency perovskite module and solar cells on 6-in. substrate using slot die coating. *Solar Energy Materials and Solar Cells* **2018**, *181*, 53–59.
21. Jiang, Y.; Leyden, M.R.; Qiu, L.; Wang, S.; Ono, L.K.; Wu, Z.; Juarez-Perez, E.J.; Qi, Y., Combination of Hybrid CVD and Cation Exchange for Upscaling Cs-Substituted Mixed Cation Perovskite Solar Cells with High Efficiency and Stability. *Advanced Functional Materials* **2018,** *28* (1), 1703835.
22. Nia, N.Y.; Zendehdel, M.; Cinà, L.; Matteocci, F.; Di Carlo, A., A crystal engineering approach for scalable perovskite solar cells and module fabrication: A full out of glove box procedure. *Journal of Materials Chemistry A* **2018,** *6* (2), 659-671.
23. Yang, M.; Kim, D.H.; Klein, T.R.; Li, Z.; Reese, M.O.; Tremolet de Villers, B.J.; Berry, J.J.; van Hest, M.F.; Zhu, K., Highly efficient perovskite solar modules by scalable fabrication and interconnection optimization. *ACS Energy Letters* **2018,** *3* (2), 322-328.
24. Li, K.; Xiao, J.; Yu, X.; Li, T.; Xiao, D.; He, J.; Zhou, P.; Zhang, Y.; Li, W.; Ku, Z., An efficient, flexible perovskite solar module exceeding 8% prepared with an ultrafast PbI 2 deposition rate. *Scientific reports* **2018,** *8* (1), 1-8.
25. Ding, B.; Huang, S.-Y.; Chu, Q.-Q.; Li, Y.; Li, C.-X.; Li, C.-J.; Yang, G.-J., Low-temperature SnO 2-modified TiO 2 yields record efficiency for normal planar perovskite solar modules. *Journal of Materials Chemistry A* **2018,** *6* (22), 10233-10242.
26. Bu, T.; Shi, S.; Li, J.; Liu, Y.; Shi, J.; Chen, L.; Liu, X.; Qiu, J.; Ku, Z.; Peng, Y., Low-temperature presynthesized crystalline tin oxide for efficient flexible perovskite solar cells and modules. *ACS applied materials & interfaces* **2018,** *10* (17), 14922-14929.
27. He, J.; Bi, E.; Tang, W.; Wang, Y.; Yang, X.; Chen, H.; Han, L., Low-temperature soft-cover-assisted hydrolysis deposition of large-scale TiO 2 layer for efficient perovskite solar modules. *Nano-micro letters* **2018,** *10* (3), 1-8.
28. Dagar, J.; Castro-Hermosa, S.; Gasbarri, M.; Palma, A.L.; Cina, L.; Matteocci, F.; Calabrò, E.; Di Carlo, A.; Brown, T.M., Efficient fully laser-patterned flexible perovskite modules and solar cells based on low-temperature solution-processed SnO 2/mesoporous-TiO 2 electron transport layers. *Nano Research* **2018,** *11* (5), 2669-2681.
29. Deng, Y.; Zheng, X.; Bai, Y.; Wang, Q.; Zhao, J.; Huang, J., Surfactant-controlled ink drying enables high-speed deposition of perovskite films for efficient photovoltaic modules. *Nature Energy* **2018,** *3* (7), 560-566.

30. De Rossi, F.; Baker, J.A.; Beynon, D.; Hooper, K.E.; Meroni, S.M.; Williams, D.; Wei, Z.; Yasin, A.; Charbonneau, C.; Jewell, E.H., All printable perovskite solar modules with 198 cm2 active area and over 6% efficiency. *Advanced Materials Technologies* **2018,** *3* (11), 1800156.
31. Zhang, F.; Cong, J.; Li, Y.; Bergstrand, J.; Liu, H.; Cai, B.; Hajian, A.; Yao, Z.; Wang, L.; Hao, Y., A facile route to grain morphology controllable perovskite thin films towards highly efficient perovskite solar cells. *Nano Energy* **2018**, *53*, 405–414.
32. Hu, X.; Huang, Z.; Li, F.; Su, M.; Huang, Z.; Zhao, Z.; Cai, Z.; Yang, X.; Meng, X.; Li, P., Nacre-inspired crystallization and elastic "brick-and-mortar" structure for a wearable perovskite solar module. *Energy & Environmental Science* **2019,** *12* (3), 979-987.
33. Li, P.; Zhang, Y.; Liang, C.; Xing, G.; Liu, X.; Li, F.; Liu, X.; Hu, X.; Shao, G.; Song, Y., Phase pure 2D perovskite for high-performance 2D–3D heterostructured perovskite solar cells. *Advanced materials* **2018,** *30* (52), 1805323.
34. Bu, T.; Li, J.; Zheng, F.; Chen, W.; Wen, X.; Ku, Z.; Peng, Y.; Zhong, J.; Cheng, Y.-B.; Huang, F., Universal passivation strategy to slot-die printed SnO 2 for hysteresis-free efficient flexible perovskite solar module. *Nature communications* **2018,** *9* (1), 1-10.
35. Jiang, Y.; Remeika, M.; Hu, Z.; Juarez-Perez, E.J.; Qiu, L.; Liu, Z.; Kim, T.; Ono, L.K.; Son, D.Y.; Hawash, Z., Negligible-Pb-Waste and Upscalable Perovskite Deposition Technology for High-Operational-Stability Perovskite Solar Modules. *Advanced Energy Materials* **2019,** *9* (13), 1803047.
36. Qiu, L.; He, S.; Jiang, Y.; Son, D.-Y.; Ono, L.K.; Liu, Z.; Kim, T.; Bouloumis, T.; Kazaoui, S.; Qi, Y., Hybrid chemical vapor deposition enables scalable and stable Cs-FA mixed cation perovskite solar modules with a designated area of 91.8 cm 2 approaching 10% efficiency. *Journal of Materials Chemistry A* **2019,** *7* (12), 6920-6929.
37. Bashir, A.; Lew, J.H.; Shukla, S.; Gupta, D.; Baikie, T.; Chakraborty, S.; Patidar, R.; Bruno, A.; Mhaisalkar, S.; Akhter, Z., Cu-doped nickel oxide interface layer with nanoscale thickness for efficient and highly stable printable carbon-based perovskite solar cell. *Solar Energy* **2019**, *182*, 225–236.
38. Tian, S.; Li, J.; Li, S.; Bu, T.; Mo, Y.; Wang, S.; Li, W.; Huang, F., A facile green solvent engineering for up-scaling perovskite solar cell modules. *Solar Energy* **2019**, *183*, 386–391.
39. Jung, E.H.; Jeon, N.J.; Park, E.Y.; Moon, C.S.; Shin, T.J.; Yang, T.-Y.; Noh, J.H.; Seo, J., Efficient, stable and scalable perovskite solar cells using poly (3-hexylthiophene). *Nature* **2019,** *567* (7749), 511-515.
40. Abuhelaiqa, M.; Paek, S.; Lee, Y.; Cho, K.T.; Heo, S.; Oveisi, E.; Huckaba, A.J.; Kanda, H.; Kim, H.; Zhang, Y., Stable perovskite solar cells using tin acetylacetonate based electron transporting layers. *Energy & Environmental Science* **2019,** *12* (6), 1910-1917.
41. Ozaki, M.; Shimazaki, A.; Jung, M.; Nakaike, Y.; Maruyama, N.; Yakumaru, S.; Rafieh, A.I.; Sasamori, T.; Tokitoh, N.; Ekanayake, P., A Purified, Solvent-Intercalated Precursor Complex for Wide-Process-Window Fabrication of Efficient Perovskite Solar Cells and Modules. *Angewandte Chemie International Edition* **2019,** *58* (28), 9389-9393.
42. Gotanda, T.; Oooka, H.; Mori, S.; Nakao, H.; Amano, A.; Todori, K.; Nakai, Y.; Mizuguchi, K., Facile and scalable fabrication of low-hysteresis perovskite solar cells and modules using a three-step process for the perovskite layer. *Journal of Power Sources* **2019**, *430*, 145–149.
43. Matteocci, F.; Vesce, L.; Kosasih, F.U.; Castriotta, L.A.; Cacovich, S.; Palma, A.L.; Divitini, G.; Ducati, C.; Di Carlo, A., Fabrication and morphological characterization of high-efficiency blade-coated perovskite solar modules. *ACS applied materials & interfaces* **2019,** *11* (28), 25195-25204.
44. Hu, X.; Meng, X.; Zhang, L.; Zhang, Y.; Cai, Z.; Huang, Z.; Su, M.; Wang, Y.; Li, M.; Li, F., A mechanically robust conducting polymer network electrode for efficient flexible perovskite solar cells. *Joule* **2019,** *3* (9), 2205-2218.
45. Bu, T.; Liu, X.; Li, J.; Huang, W.; Wu, Z.; Huang, F.; Cheng, Y.-B.; Zhong, J., Dynamic antisolvent engineering for spin coating of 10× 10 cm2 perovskite solar module approaching 18%. *Solar RRL* **2020,** *4* (2), 1900263.
46. Lou, L.; Liu, T.; Xiao, J.; Xiao, S.; Long, X.; Zheng, S.; Yang, S., Controlling Apparent Coordinated Solvent Number in the Perovskite Intermediate Phase Film for Developing Large-Area Perovskite Solar Modules. *Energy Technology* **2020,** *8* (4), 1900972.
47. Dai, X.; Deng, Y.; Van Brackle, C.H.; Chen, S.; Rudd, P.N.; Xiao, X.; Lin, Y.; Chen, B.; Huang, J., Scalable fabrication of efficient perovskite solar modules on flexible glass substrates. *Advanced Energy Materials* **2020,** *10* (1), 1903108.
48. Qiu, L.; Liu, Z.; Ono, L.K.; Jiang, Y.; Son, D.Y.; Hawash, Z.; He, S.; Qi, Y., Scalable fabrication of stable high efficiency perovskite solar cells and modules utilizing room temperature sputtered SnO2 electron transport layer. *Advanced Functional Materials* **2019,** *29* (47), 1806779.
49. Kwon, H.-C.; Ma, S.; Yun, S.-C.; Jang, G.; Yang, H.; Moon, J., A nanopillar-structured perovskite-based efficient semitransparent solar module for power-generating window applications. *Journal of Materials Chemistry A* **2020,** *8* (3), 1457-1468.
50. Nia, N.Y.; Giordano, F.; Zendehdel, M.; Cinà, L.; Palma, A.L.; Medaglia, P.G.; Zakeeruddin, S.M.; Grätzel, M.; Di Carlo, A., Solution-based heteroepitaxial growth of stable mixed cation/anion hybrid perovskite thin film under ambient condition via a scalable crystal engineering approach. *Nano Energy* **2020**, *69*, 104441.
51. Liu, C.; Yang, Y.; Zhang, C.; Wu, S.; Wei, L.; Guo, F.; Arumugam, G.M.; Hu, J.; Liu, X.; Lin, J., Tailoring C60 for efficient inorganic CsPbI2Br perovskite solar cells and modules. *Advanced Materials* **2020,** *32* (8), 1907361.
52. Meng, X.; Cai, Z.; Zhang, Y.; Hu, X.; Xing, Z.; Huang, Z.; Huang, Z.; Cui, Y.; Hu, T.; Su, M., Bio-inspired vertebral design for scalable and flexible perovskite solar cells. *Nature communications* **2020,** *11* (1), 1-10.
53. Ren, A.; Lai, H.; Hao, X.; Tang, Z.; Xu, H.; Jeco, B.M.F.Y.; Watanabe, K.; Wu, L.; Zhang, J.; Sugiyama, M., Efficient perovskite solar modules with minimized nonradiative recombination and local carrier transport losses. *Joule* **2020,** *4* (6), 1263-1277.
54. Taheri, B.; Calabrò, E.; Matteocci, F.; Di Girolamo, D.; Cardone, G.; Liscio, A.; Di Carlo, A.; Brunetti, F. Automated scalable spray coating of SnO2 for the fabrication of low-temperature perovskite solar cells and modules. *Energy Technology* **2020,** *8* (5), 1901284.

55. Raptis, D.; Stoichkov, V.; Meroni, S.M.; Pockett, A.; Worsley, C.A.; Carnie, M.; Worsley, D.A.; Watson, T., Enhancing fully printable mesoscopic perovskite solar cell performance using integrated metallic grids to improve carbon electrode conductivity. *Current Applied Physics* **2020,** *20* (5), 619-627.
56. Huang, X.; Chen, R.; Deng, G.; Han, F.; Ruan, P.; Cheng, F.; Yin, J.; Wu, B.; Zheng, N., Methylamine-Dimer-Induced Phase Transition toward MAPbI3 Films and High-Efficiency Perovskite Solar Modules. *Journal of the American Chemical Society* **2020,** *142* (13), 6149-6157.
57. Meroni, S.M.; Hooper, K.E.; Dunlop, T.; Baker, J.A.; Worsley, D.; Charbonneau, C.; Watson, T.M., Scribing Method for Carbon Perovskite Solar Modules. *Energies* **2020,** *13* (7), 1589.
58. Li, J.; Wang, H.; Chin, X.Y.; Dewi, H.A.; Vergeer, K.; Goh, T.W.; Lim, J.W.M.; Lew, J.H.; Loh, K.P.; Soci, C., Highly efficient thermally co-evaporated perovskite solar cells and mini-modules. *Joule* **2020,** *4* (5), 1035-1053.
59. Zhang, J.; Bu, T.; Li, J.; Li, H.; Mo, Y.; Wu, Z.; Liu, Y.; Zhang, X.-L.; Cheng, Y.-B.; Huang, F., Two-step sequential blade-coating of high quality perovskite layers for efficient solar cells and modules. *Journal of Materials Chemistry A* **2020,** *8* (17), 8447-8454.
60. Lim, K.-S.; Lee, D.-K.; Lee, J.-W.; Park, N.-G., 17% efficient perovskite solar mini-module via hexamethylphosphoramide (HMPA)-adduct-based large-area D-bar coating. *Journal of Materials Chemistry A* **2020,** *8* (18), 9345-9354.
61. Chen, R.; Hui, Y.; Wu, B.; Wang, Y.; Huang, X.; Xu, Z.; Ruan, P.; Zhang, W.; Cheng, F.; Zhang, W., Moisture-tolerant and high-quality α-CsPbI 3 films for efficient and stable perovskite solar modules. *Journal of Materials Chemistry A* **2020,** *8* (19), 9597-9606.
62. Xu, M.; Ji, W.; Sheng, Y.; Wu, Y.; Cheng, H.; Meng, J.; Yan, Z.; Xu, J.; Mei, A.; Hu, Y., Efficient triple-mesoscopic perovskite solar mini-modules fabricated with slot-die coating. *Nano Energy* **2020**, *74*, 104842.
63. Lee, K.-M.; Lai, C.-H.; Chu, W.-C.; Chan, S.-H.; Suryanarayanan, V., Thermal assisted blade coating methylammonium lead iodide films with non-toxic solvent precursors for efficient perovskite solar cells and sub-module. *Solar Energy* **2020**, *204*, 337–345.
64. Wang, C.; Tan, G.; Luo, X.; Li, J.; Gao, X.; Mo, Y.; Zhang, X.-L.; Wang, X.; Huang, F., How to fabricate efficient perovskite solar mini-modules in lab. *Journal of Power Sources* **2020**, *466*, 228321.
65. Zhang, Y.; Chen, M.; Zhou, Y.; Li, W.; Lee, Y.; Kanda, H.; Gao, X.X.; Hu, R.; Brooks, K.G.; Zia, R., The Synergism of DMSO and Diethyl Ether for Highly Reproducible and Efficient MA0. 5FA0. 5PbI3 Perovskite Solar Cells. *Advanced Energy Materials* **2020,** *10* (29), 2001300.
66. Lei, T.; Li, F.; Zhu, X.; Dong, H.; Niu, Z.; Ye, S.; Zhao, W.; Xi, J.; Jiao, B.; Ding, L., Flexible perovskite solar modules with functional layers fully vacuum deposited. *Solar RRL* **2020,** *4* (11), 2000292.
67. Qiu, L.; He, S.; Liu, Z.; Ono, L.K.; Son, D.-Y.; Liu, Y.; Tong, G.; Qi, Y., Rapid hybrid chemical vapor deposition for efficient and hysteresis-free perovskite solar modules with an operation lifetime exceeding 800 hours. *Journal of Materials Chemistry A* **2020,** *8* (44), 23404-23412.
68. Du, M.; Zhu, X.; Wang, L.; Wang, H.; Feng, J.; Jiang, X.; Cao, Y.; Sun, Y.; Duan, L.; Jiao, Y., High-Pressure Nitrogen-Extraction and Effective Passivation to Attain Highest Large-Area Perovskite Solar Module Efficiency. *Advanced Materials* **2020,** *32* (47), 2004979.
69. Chung, J.; Shin, S.S.; Hwang, K.; Kim, G.; Kim, K.W.; Kim, W.; Ma, B.S.; Kim, Y.-K.; Kim, T.-S.; Seo, J., Record-efficiency flexible perovskite solar cell and module enabled by a porous-planar structure as an electron transport layer. *Energy & Environmental Science* **2020,** *13* (12), 4854-4861.
70. Nia, N.Y.; Zendehdel, M.; Abdi-Jalebi, M.; Castriotta, L.A.; Kosasih, F.U.; Lamanna, E.; Abolhasani, M.M.; Zheng, Z.; Andaji-Garmaroudi, Z.; Asadi, K., Beyond 17% stable perovskite solar module via polaron arrangement of tuned polymeric hole transport layer. *Nano Energy* **2021**, *82*, 105685.
71. Rolston, N.; Scheideler, W.J.; Flick, A.C.; Chen, J.P.; Elmaraghi, H.; Sleugh, A.; Zhao, O.; Woodhouse, M.; Dauskardt, R.H., Rapid open-air fabrication of perovskite solar modules. *Joule* **2020,** *4* (12), 2675-2692.
72. Yang, J.; Tang, W.; Yuan, R.; Chen, Y.; Wang, J.; Wu, Y.; Yin, W.-J.; Yuan, N.; Ding, J.; Zhang, W.-H., Defect mitigation using d-penicillamine for efficient methylammonium-free perovskite solar cells with high operational stability. *Chemical Science* **2021,** *12* (6), 2050-2059.
73. Lee, D.-K.; Lim, K.-S.; Lee, J.-W.; Park, N.-G., Scalable perovskite coating via anti-solvent-free Lewis acid–base adduct engineering for efficient perovskite solar modules. *Journal of Materials Chemistry A* **2021,** *9* (5), 3018-3028.
74. Chen, R.; Wu, Y.; Wang, Y.; Xu, R.; He, R.; Fan, Y.; Huang, X.; Yin, J.; Wu, B.; Li, J., Crown Ether-Assisted Growth and Scaling Up of FACsPbI3 Films for Efficient and Stable Perovskite Solar Modules. *Advanced Functional Materials* **2021,** *31* (11), 2008760.
75. Sha, Y.; Bi, E.; Zhang, Y.; Ru, P.; Kong, W.; Zhang, P.; Yang, X.; Chen, H.; Han, L., A Scalable Integrated Dopant-Free Heterostructure to Stabilize Perovskite Solar Cell Modules. *Advanced Energy Materials* **2021,** *11* (5), 2003301.
76. Wang, H.; Huang, Z.; Xiao, S.; Meng, X.; Xing, Z.; Rao, L.; Gong, C.; Wu, R.; Hu, T.; Tan, L., An in situ bifacial passivation strategy for flexible perovskite solar module with mechanical robustness by roll-to-roll fabrication. *Journal of Materials Chemistry A* **2021,** *9* (9), 5759-5768.
77. Tong, G.; Son, D.Y.; Ono, L.K.; Liu, Y.; Hu, Y.; Zhang, H.; Jamshaid, A.; Qiu, L.; Liu, Z.; Qi, Y., Scalable Fabrication of> 90 cm2 Perovskite Solar Modules with> 1000 h Operational Stability Based on the Intermediate Phase Strategy. *Advanced Energy Materials* **2021,** *11* (10), 2003712.
78. Castriotta, L.A.; Matteocci, F.; Vesce, L.; Cinà, L.; Agresti, A.; Pescetelli, S.; Ronconi, A.; Löffler, M.; Stylianakis, M.M.; Di Giacomo, F., Air-Processed Infrared-Annealed Printed Methylammonium-Free Perovskite Solar Cells and Modules Incorporating Potassium-Doped Graphene Oxide as an Interlayer. *ACS Applied Materials & Interfaces* **2021,** *13* (10), 11741-11754.

79. Taheri, B.; De Rossi, F.; Lucarelli, G.; Castriotta, L.A.; Di Carlo, A.; Brown, T.M.; Brunetti, F., Laser-Scribing Optimization for Sprayed SnO2-Based Perovskite Solar Modules on Flexible Plastic Substrates. *ACS Applied Energy Materials* **2021,** *4* (5), 4507-4518.
80. Dagar, J.; Fenske, M.; Al-Ashouri, A.; Schultz, C.; Li, B.; Köbler, H.; Munir, R.; Parmasivam, G.; Li, J.; Levine, I., Compositional and Interfacial Engineering Yield High-Performance and Stable pin Perovskite Solar Cells and Mini-Modules. *ACS applied materials & interfaces* **2021,** *13* (11), 13022-13033.
81. Kim, Y.-C.; Heo, Y.-J.; Lee, S.K.; Jung, Y.-S.; Jeong, H.-J.; Kim, S.-T.; Yoo, J.-S.; Kim, D.-Y.; Jang, J.-H., Improved light absorption in perovskite solar module employing nanostructured micro-prism array. *Solar Energy Materials and Solar Cells* **2021**, *226*, 111077.
82. Chen, S.; Deng, Y.; Xiao, X.; Xu, S.; Rudd, P.N.; Huang, J., Preventing lead leakage with built-in resin layers for sustainable perovskite solar cells. *Nature Sustainability* **2021**, 1-8.
83. Yang, Z.; Zhang, W.; Wu, S.; Zhu, H.; Liu, Z.; Liu, Z.; Jiang, Z.; Chen, R.; Zhou, J.; Lu, Q., Slot-die coating large-area formamidinium-cesium perovskite film for efficient and stable parallel solar module. *Science Advances* **2021,** *7* (18), eabg3749.
84. Chen, S.; Xiao, X.; Gu, H.; Huang, J., Iodine reduction for reproducible and high-performance perovskite solar cells and modules. *Science Advances* **2021,** *7* (10), eabe8130.
85. Tong, G.; Son, D.-Y.; Ono, L.K.; Kang, H.-B.; He, S.; Qiu, L.; Zhang, H.; Liu, Y.; Hieulle, J.; Qi, Y., Removal of residual compositions by powder engineering for high efficiency formamidinium-based perovskite solar cells with operation lifetime over 2000 h. *Nano Energy* **2021**, *87*, 106152.
86. Yang, F.; Dong, L.; Jang, D.; Saparov, B.; Tam, K.C.; Zhang, K.; Li, N.; Brabec, C.J.; Egelhaaf, H.J., Low Temperature Processed Fully Printed Efficient Planar Structure Carbon Electrode Perovskite Solar Cells and Modules. *Advanced Energy Materials* **2021**, 2101219.
87. Castriotta, L.A.; Fuentes Pineda, R.; Babu, V.; Spinelli, P.; Taheri, B.; Matteocci, F.; Brunetti, F.; Wojciechowski, K.; Di Carlo, A., Light-Stable Methylammonium-Free Inverted Flexible Perovskite Solar Modules on PET Exceeding 10.5% on a 15.7 cm2 Active Area. *ACS Applied Materials & Interfaces* **2021**.
88. Chen, R.; Wang, Y.; Nie, S.; Shen, H.; Hui, Y.; Peng, J.; Wu, B.; Yin, J.; Li, J.; Zheng, N., Sulfonate-Assisted Surface Iodide Management for High-Performance Perovskite Solar Cells and Modules. *Journal of the American Chemical Society* **2021,** *143* (28), 10624-10632.
89. Zhu, J.; Park, S.; Gong, O.Y.; Sohn, C.; Li, Z.; Zhang, Z.; Jo, B.; Kim, W.; Han, G.S.; Kim, D.H., Formamidine disulfide oxidant as a localised electron scavenger for> 20% perovskite solar cell modules. *Energy & Environmental Science* **2021**.
90. Tong, G.; Ono, L.K.; Liu, Y.; Zhang, H.; Bu, T.; Qi, Y., Up-Scalable Fabrication of SnO2 with Multifunctional Interface for High Performance Perovskite Solar Modules. *Nano-Micro Letters* **2021,** *13* (1), 1-14.
91. Liu, Z.; Qiu, L.; Ono, L.K.; He, S.; Hu, Z.; Jiang, M.; Tong, G.; Wu, Z.; Jiang, Y.; Son, D.-Y., A holistic approach to interface stabilization for efficient perovskite solar modules with over 2,000-hour operational stability. *Nature Energy* **2020,** *5* (8), 596-604.
92. Wang, Y.; Ju, H.; Mahmoudi, T.; Liu, C.; Zhang, C.; Wu, S.; Yang, Y.; Wang, Z.; Hu, J.; Cao, Y., Cation-size mismatch and interface stabilization for efficient NiOx-based inverted perovskite solar cells with 21.9% efficiency. *Nano Energy* **2021**, *88*, 106285.
